# On the emergence of the correlation between life expectancy and the variance in the age at death

**DOI:** 10.1098/rsos.220020

**Published:** 2022-11-09

**Authors:** Oscar E. Fernandez, Hiram Beltrán-Sánchez

**Affiliations:** ^1^ Department of Mathematics, Wellesley College, Wellesley, MA 02482, USA; ^2^ Fielding School of Public Health and California Center for Population Research, UCLA, Los Angeles, CA, USA

**Keywords:** mortality, mortality compression, lifespan inequality

## Abstract

Recent empirical studies have found various patterns in the correlations between lifespan inequality and life expectancy in modern human populations. However, it is unclear how general these regularities are. Here we establish three theorems that provide theoretical foundations for such regularities. We show that for populations with a finite maximum lifespan *ω*, and under certain continuity assumptions, the variance in the age at death is bounded by a function of lifespan that has a maximum and tends to zero as life expectancy tends to zero and *ω*. We show how the change in said variance is determined by a particular interplay between the coefficient of variation and the mean age in the population. These results lead to three hypotheses—a three-phased pattern of change for the correlation between the variance and life expectancy, a particular shape of the associated variance function, and that survival curve Type is one driver of the pattern. We illustrate those hypotheses empirically via a study of the 10 countries in the Human Mortality Database with the oldest available data. Our results elucidate the emergence of the aforementioned correlation patterns and provide demographically meaningful conditions under which those correlations reverse.

## 1. Introduction

Over the past several decades, there has been a renewed interest in the interplay between lifespan inequality and life expectancy in wild and human populations. In humans, for example, it is a well-documented phenomenon that social health risk factors (e.g. social determinants of health) and social conditions operate as fundamental causes of disease and death (see, e.g. [1–3] and references therein) and underlie the variability in ages at death in a population whereby, for example, individuals with low socio-economic status and/or with social disadvantage tend to die at younger ages than those in more privileged positions [4]. While demographers use a variety of metrics to quantify this variability in lifespans (see [5] and references therein for a summary), two of the most commonly used metrics are the variance in the ages at death, *σ*^2^, and the ‘lifespan disparity’ [6–10], $e^{†}$. These metrics’ useful demographic interpretations are what probably drive their popularity: the latter ($e^{†}$) has been shown to measure the average life expectancy lost due to death [9,10] and is intimately related to other information-theoretic lifespan inequality metrics (see [11] for a discussion); the former (*σ*^2^) is a more traditional statistical measure of dispersion that describes the age-wise spread of mortality in the population’s deaths distribution. Empirical work using these lifespan inequality metrics finds that in modern human populations both $e^{†}$ and *σ*^2^ are positively correlated with life expectancy at birth (*e*_0_) for $e_{0}⪅40\;\mathrm{years}$, uncorrelated for $e_{0}\approx 40\;\mathrm{years}$, and negatively correlated for $e_{0}>40\;\mathrm{years}$ [12–16]. One ramification of this particular three-phased pattern of correlation is that $e^{†}$ and *σ*^2^ are positively correlated, a well-known empirical finding that was only recently partially explained theoretically by the work of Fernandez & Beltrán-Sánchez [17], which shows that $e^{†}$ can be expanded in a series whose terms are proportional to the central moments of the deaths distribution, with the lowest-order term in the expansion proportional to *σ*^2^. But why do both $e^{†}$ and *σ*^2^ follow the particular three-phased pattern of change in their correlation with *e*_0_? And does this regularity extend beyond modern human populations and hold for other species?

One way to investigate these questions would be to continue the approach taken in the empirical studies cited thus far. But this is infeasible—there are simply too many species in the tree of life. A more tractable approach is to undertake a theoretical investigation of how lifespan inequality and life expectancy interrelate. Some work in this direction has already been done and has successfully linked lifespan inequality metrics to *e*_0_. However, the vast majority of the available results to date hold only for certain parametric mortality models and under an infinite maximum lifespan assumption (e.g. [18] relates the ‘life table entropy’ $H=e^{†}/e_{0}$—another metric of lifespan inequality—to *e*_0_ under a Gompertz mortality model and assuming an infinite maximum lifespan). While such parametric models may be accurate for certain populations they will not be accurate for all. Additionally, the assumption that the maximum lifespan is infinite (a common assumption in theoretical demography), while convenient for theoretical analyses, is problematic from an empirical standpoint. (How would one verify, empirically, that a species’ maximum lifespan is infinite?) To our knowledge, only one article [19] contains some theoretical results on the relationship between lifespan inequality and life expectancy in the setting of a finite maximum lifespan. This underscores the need for additional research into the two questions asked at the end of the previous paragraph in the setting of finite maximum lifespan.

This article therefore addresses said open questions by conducting a theoretical investigation of how the variance in the age at death changes in response to changes in life expectancy. Our aim is to make the minimal set of assumptions that yields the greatest insight into the origins of the empirical regularities cited above. Using fairly straightforward mathematics, we employ this approach and establish foundational results that have implications for the shape of the variance functions—the curves of the variance of the deaths distribution as a function of life expectancy—for real-world populations with finite maximum lifespans and provide conditions for when this variance increases or decreases. We deduce from these theoretical results three hypotheses describing the shape of these variance curves. The first, the Variance Curve Correlation Hypothesis, posits that the correlation between the variance in the age at death and life expectancy changes from positive to zero to negative as life expectancy ranges from zero to the maximum lifespan in the population; this hypothesis extends, to a general species with a finite maximum lifespan, the three-phased correlation pattern we discussed earlier that has been empirically documented in modern humans. The second hypothesis, the Variance Curve Type Hypothesis, links these three correlation phases to the population’s survival curve types [20–22]. The final hypothesis, the Variance Curve Pattern Hypothesis, posits that the curves describing the variance functions in real-world populations are small perturbations of a ‘fundamental variance curve’ (which we describe in more detail).

The article is organized as follows. In §2, we present three theoretical results that underpin the rest of our work. Theorem 2.1 establishes an upper bound for the variance of ages at death in terms of the life expectancy at birth; theorems 2.4 and 2.5 describe how that variance changes in response to finite, infinitesimal and functional changes in life expectancy, and provide conditions for when the variance–life expectancy rate of change is positive or negative. In §3, we extract from these results our three hypotheses. (For interested readers, we illustrate all these results analytically in appendix B using a hyperbolic mortality model.) We investigate the hypotheses empirically in §4, where we present empirical support for them and our theoretical results across the 10 countries in the Human Mortality Database [23] with the oldest data. Finally, in §5, we discuss some extensions of our findings, explore the potential biological and ecological drivers of our results via connections between our results and the fast–slow continuum well known in animals [24], and propose additional directions for future research.

## 2. Linking changes in life expectancy to changes in the variance of the deaths distribution

Consider an age-structured population with finite maximum lifespan *ω*—let us refer to such populations as *terminal populations*—and denote by *x* the age of members in the population (measured in years). In theoretical demography sometimes *ω* = ∞ is considered. However, the presentation of our results below requires a finite *ω*-value. Whether—and to what extent—our results herein generalize to the *ω* = ∞ setting is an open question, and not the main objective of our investigation. As we articulated earlier, our main objective is to establish those results themselves, of which the most notable is a theoretical study of how the empirically documented variance–life expectancy correlation phases emerge. And, as with any theoretical study, ours necessarily requires making assumptions, the chief one of which in our case is that *ω* is finite. We will discuss this assumption further in §4 and in the Conclusion, after we have presented the findings we show flow from this assumption.

The *distribution of ages at death* (*deaths distribution*, for short) in the population, which we will denote by *f*(*x*), encodes information about how aggregate mortality is distributed across age in the population. We assume hereafter that this distribution is constructed from life tables. Denoting by *μ*(*x*) the *force of mortality* function, the instantaneous rate of death at age *x* conditioned upon surviving to that age, we have [25]

$$\mu (x)=-{\left[\ln(s(x))\right]}^{\prime },$$

where *s*(*x*) is the population’s *survival function* and the apostrophe denotes differentiation with respect to age (*x*). The deaths distribution is then defined as

2.1 $$f\;(x)=\mu (x)s(x)=-s^{\prime }(x).$$

The mean value of *f* is well known to demographers as the *life expectancy at birth*,

2.2 $$e_{0}=\int_{0}^{\omega }xf\;(x)\;dx=\int_{0}^{\omega }s(x)\;dx.$$

A natural measure of the variation in this life expectancy is the variance in the ages at death,

2.3 $$\sigma^{2}=\int_{0}^{\omega }{\left(x-e_{0}\right)}^{2}f\;(x)\;dx=\int_{0}^{\omega }x^{2}f\;(x)\;dx-e_{0}^{2}.$$

(We assume hereafter that all the life table functions named above possess the required properties to ensure that *e*_0_ and *σ*^2^ exist.) The presence of *e*_0_ in this equation suggests that perhaps there is some analytical relationship between the variance *σ*^2^ and the life expectancy *e*_0_. Indeed, empirical evidence for such relationships (between *σ* and *e*_0_, specifically) have been documented in humans [14,15]. The first question we would like to investigate, therefore, is: how does *σ*^2^ depend on *e*_0_ for terminal populations? An important step toward answering that question is provided by the following theorem.

**Theorem 2.1.**

*Let σ*^2^, *e*_0_ *and ω denote the variance in ages at death*, *life expectancy at birth and maximum lifespan in a terminal population. Then the following inequality holds*:

2.4 $$0\leq \sigma^{2}\leq \frac{\omega^{2}}{4}-{\left(e_{0}-\frac{\omega }{2}\right)}^{2}.$$

(See appendix A for the proof of the theorem above. We note also that this theorem was independently derived in [19] using methods different from our own; see also [19] for similar results for other lifespan inequality metrics.)

Theorem 2.1 says that given a terminal population (and thus an *ω*-value) and its deaths distribution *f*, assuming (as we have) that *f* has the requisite properties to ensure that *e*_0_ and *σ*^2^ exist, then (2.4) must hold. In other words, in terminal populations (with well-defined *e*_0_- and *σ*^2^-values) the variance of the deaths distribution is bounded above by a (quadratic) function of the population’s life expectancy. If we fix the *ω*-value and now imagine the population’s deaths distribution *f* changing over time, then theorem 2.1 applies at each stage and the upper bound in (2.4) begins to trace out the graph of the quadratic function of *e*_0_, *ψ*(*e*_0_) = (*ω*^2^/4) − (*e*_0_ − (*ω*/2))^2^, the right-hand side of (2.4). This leads to a useful graphical understanding of (2.4) that we now describe in more detail.

**Figure 1.**
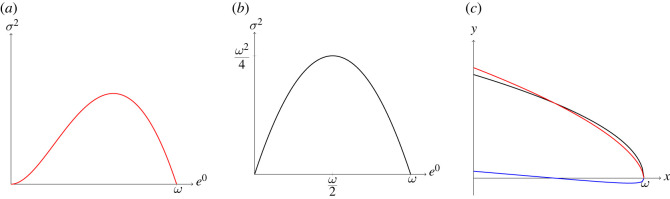
(*a*) An example of a variance curve. (*b*) The graph of the function *σ*^2^(*e*_0_) = *ω*^2^/4 − (*e*_0_ − *ω*/2)^2^, the upper bound in (2.4). (*c*) Plots of the deaths distributions *y* = *f*(*x*) = (1.5/*ω*)(1 − (*x*/*ω*))^0.5^ (black) and *y* = *g*(*x*) = (1.6/*ω*)(1 − (*x*/*ω*))^0.6^ (red), and the difference *y* = *g*(*x*) − *f*(*x*) (blue).

We note first that the values of *e*_0_ and *σ*^2^ calculated from *f* at some time *t* via (2.2)–(2.3) generate the ordered pair (*e*_0_(*t*), *σ*^2^(*t*)). We can plot this as a point in *e*_0_–*σ*^2^ space, a space akin to a ‘pace-shape’ space [26] and also reminiscent of the phase space of a mechanical system. As *t* ranges over some interval [*a*, *b*] we generate the parametric function *γ*(*t*) = (*e*_0_(*t*), *σ*^2^(*t*)). We will call the graph of *γ* (which we denote by *G*(*γ*)), the plot in *e*_0_–*σ*^2^ space of all points in the set {(*e*_0_(*t*), *σ*^2^(*t*)) : *t* ∈ [*a*, *b*]}, the *variance curve* of the terminal population. Figure 1*a* shows a hypothetical continuous variance curve. Of course, not all variance curves are continuous, and not all variance curves are graphs of a function of *e*_0_. (This is one reason why we have chosen to define variance curves parametrically—such a definition is general enough to model variance curves with self-intersections, which may be generated from real-world data if *e*_0_(*t*_1_) = *e*_0_(*t*_2_) yet *σ*^2^(*t*_1_) ≠ *σ*^2^(*t*_2_) for *t*_1_ ≠ *t*_2_.)

Now, if we envision using non-continuous data (e.g. life-table data for a real-world population) to generate *G*(*γ*), our plot of (*e*_0_(*t*), *σ*^2^(*t*)) for each successive *t*-value in the dataset would generate points that jump around in *e*_0_–*σ*^2^ space, with some doubling back to regions containing points already plotted (as when *e*_0_(*t*_2_) < *e*_0_(*t*_1_) for *t*_2_ > *t*_1_). Once all points in the dataset are plotted and we obtain *G*(*γ*), what would result is the typical scatterplot of data contained in many of the empirical studies referenced in the Introduction (skip ahead to figure 3, blue data, for an example of this). Our earlier question about the dependency of *σ*^2^ on *e*_0_ can then be broken into two different questions, depending on whether one wishes to retain information about *t* or not: what is the rate of change of *σ*^2^ with respect to *e*_0_? What is the shape of the variance curve *G*(*γ*)? The first question retains some interest in the parameter *t*, while the second ignores the parameter *t* and encourages, for example, the identification of patterns (like correlations) in the dataset. (A similar situation occurs in the qualitative study of plane autonomous differential equations and their orbits.) Indeed, it is this last viewpoint that is employed in the studies mentioned in the Introduction that document the three-phased pattern of variance–life expectancy correlation in modern human populations. For this reason, let us first see what more our parametric approach can say about the shape of *G*(*γ*) and later return to the rate of change question.

The simplest candidates—from a mathematical standpoint—would be lines. But theorem 2.1 rules out most of those lines: terminal populations’ variance curves cannot be positively sloped lines, negatively sloped lines, or non-zero horizontal lines—all would violate (2.4). The line generated by *σ*^2^(*t*) = 0 for all *t*-values is still allowed, but we will exclude this unrealistic variance curve from consideration. For future reference, we record this assumption as assumption (A 1).

(A 1) *Non-identically zero variance curves*. We assume that all variance curves contain at least one positive variance value.

In addition, to simplify our subsequent analysis we hereafter assume that variance curves are continuous and have intervals of increase and decrease, and record this as assumption (A 2).

(A 2) *Continuous variance curves featuring intervals of increase and decrease.* We assume that all variance curves are continuous and have intervals of increase and decrease.

(We note that variance curves generated from discrete data can be interpolated to yield continuous variance curves.)

Assumption (A 1) and theorem 2.1 imply that variance curves must have curvature. We now show that assumption (A 2) implies they must have an absolute maximum value.

**Proposition 2.2.**

*Let P be a terminal population whose variance curve G*(*γ*) *is continuous and not identically zero. Then G*(*γ*) *has a maximum value*.

**Proof.**

The continuity assumption (A 2) implies that *e*_0_(*t*) and *σ*^2^(*t*) are continuous functions. It follows by the Extreme Value Theorem that each of these functions attains a maximum (and minimum) value on the interval [0, *ω*]. ▪

Our results thus far provide us with some global understanding of the shape of terminal populations’ variance curves. We now shift emphasis to study the local shape of variance curves, in particular how *σ*^2^ changes in response to small changes in *e*_0_. To do so, we explore three analytical methods for investigating such changes: finite, infinitesimal and functional.

Our first two approaches investigate the finite and infinitesimal changes in *σ*^2^ driven by a change in *e*_0_. (See appendix A for the proofs of the two theorems presented below.) Both theorems make use of the mean age $\bar{x}$ in the stationary population embedded in the corresponding life table,

2.5 $$\bar{x}=\frac{1}{e_{0}}\int_{0}^{\omega }xs(x)\;dx,$$

and involve special quantities we call *direction indicators*, which we now define.

**Definition 2.3.**

Let *f* and *g* ≠ *f* be two distributions of ages at death for a terminal population with maximum lifespan *ω*. Let *e*_0_ and *e*_0,*g*_ denote the respective life expectancies at birth, *σ*^2^ and $\sigma_{g}^{2}$ denote the respective variances of the associated deaths distributions, and $\bar{x}$ and ${\bar{x}}_{g}$ the respective mean ages in the stationary population. Denote by $\Delta (\sigma^{2})=\sigma_{g}^{2}-\sigma^{2}$, Δ*e*_0_ = *e*_0,*g*_ − *e*_0_, and $\Delta \bar{x}={\bar{x}}_{g}-\bar{x}$. Assuming that Δ*e*_0_ ≠ 0, we then define the following:

(i) We define the *discrete direction indicator I* as
  2.6 $$I\;:=\frac{\Delta \bar{x}}{\Delta e_{0}}-\frac{1}{2}(1-v^{2})+\frac{1}{2e_{0}}[2\Delta \bar{x}-\Delta e_{0}],$$
  where *v* = *σ*/*e*_0_ is the coefficient of variation.
(ii) We define the *infinitesimal direction indicator*
  2.7 $$J\;:=\frac{d\bar{x}}{de_{0}}-\frac{1}{2}(1-v^{2}).$$

At first sight these indicators appear to be a meaningless convoluted mix of demographically relevant parameters. But the theorem below gives them meaning, as we discuss afterward.

**Theorem 2.4.**

*Suppose that the hypotheses of definition 2.3 hold. Then*:

(a) *The variance–life expectancy discrete rate of change* Δ(*σ*^2^)/Δ*e*_0_ *is related to I as follows*:
  2.8 $$\frac{\Delta (\sigma^{2})}{\Delta e_{0}}=2e_{0}I.$$
  *Furthermore, after a change* Δ*e*_0_ ≠ 0 *in e*_0_, *the variance σ*^2^: (*i*) *increases if and only if I* > 0; (*ii*) *remains unchanged if and only if I* = 0; *and (iii) decreases if and only if I* < 0.
(b) *The instantaneous rate of change of the variance with respect to the life expectancy at birth*, d*σ*^2^/d*e*_0_, *is related to J as follows*:
  2.9 $$\frac{d\sigma^{2}}{de_{0}}=2e_{0}J.$$
  *Furthermore, the variance σ*^2^: *(i) increases if and only if J* > 0; *(ii) remains unchanged if and only if J* = 0; *and (iii) decreases if and only if J* < 0.

Let us now discuss three insights this theorem furnishes. First, we return to the meaning of the indicators *I* and *J*. Equations (2.8) and (2.9) show that *I* and *J* are one-half the relative change of the variance with respect to life expectancy. Each indicator, therefore, can be thought of as a normalized rate of change (of *σ*^2^ with respect to *e*_0_).

Secondly, we note that since the indicators are constructed out of demographically meaningful parameters—like the mean age in the stationary population and the coefficient of variation—they provide us with demographically meaningful conditions that explain when each of the three possible directional changes in the variance in the age at death in a terminal population materialize. For instance, if

2.10 $$\frac{d\bar{x}}{de_{0}}>\frac{1}{2}(1-v^{2}),$$

then *J* > 0 and so d*σ*^2^/d*e*_0_ > 0 via (2.9). Thus, for example, in terminal populations characterized by *σ* > *e*_0_ (equivalently, *v* > 1)—in other words, populations with fairly high death uncertainty compared with life expectancy at birth—when life expectancy increases, the variance in the age at death will also increase if the mean age in the stationary population increases. Similarly, suppose instead that the inequality sign in (2.10) is reversed, so that *J* < 0 and thus d*σ*^2^/d*e*_0_ < 0 via (2.9). Then we can say that in terminal populations characterized by *σ* < *e*_0_ (equivalently, *v* < 1)—in other words, populations with fairly low death uncertainty compared with life expectancy at birth—when life expectancy increases, the variance in the age at death will decrease if the mean age in the stationary population decreases.

Finally, we note that beyond measuring the *slopes* of variance curves, (2.8)–(2.9) can also be leveraged to understand their *concavity* and any associated inflection points. This is information that helps us quantify the curvature of variance curves. To illustrate this, consider a terminal population for which *e*_0_ increases over time. If *I* and *J* were to remain constant and both positive, for example, then (2.8)–(2.9) would imply increasing rates of change of *σ*^2^ with respect to *e*_0_, and therefore a convex variance curve. If *I* and *J* were to again both remain positive but decrease much faster than the increase in *e*_0_, for example, then (2.8)–(2.9) would imply decreasing rates of change of *σ*^2^ with respect to *e*_0_, and therefore a concave variance curve. We invite the interested reader to consult appendix B for an explicit illustration of these ideas for the theoretical example of a hyperbolic mortality case.

Our final approach to investigating changes in *σ*^2^ makes use of the calculus of variations and functional derivatives. (A note to the reader: you may skip this content without jeopardizing your understanding of the rest of the article.) We first view *e*_0_ and *σ*^2^ as functionals of the deaths density *f*, which we can do because of (2.2)–(2.3). Then, we define a variation of *f*(*x*)—denoted *δf*(*x*)—by *ɛν*(*x*), where *ν* is a function of *x* that is as smooth as *f* is and is such that *f* + *δf* is again a deaths density. (Intuitively, *f*(*x*) + *δf*(*x*) is another deaths density that closely resembles *f*(*x*); figure 1*c* illustrates this with *f* the black curve, *δf* the blue curve and *f* + *δf* the red curve.) We then examine, to first order in *ɛ*, the change *σ*^2^[*f* + *δf*] − *σ*^2^[*f*]. This yields the first variation *δ*(*σ*^2^)[*f*; *ν*], the functional analogue of the derivative of *σ*^2^[*f*] ‘in the direction of’ *ν*. (We refer the interested reader to [11] and references therein for a review of functional derivatives and the calculus of variations in the context of mathematical demography.) We then have the following result.

**Theorem 2.5.**

*Let f be the distribution of ages at death in a terminal population with maximum lifespan ω*, *let e*_0_ *and σ*^2^ *be the associated life expectancy at birth and variance, respectively, and let δf*(*x*) = *ɛν*(*x*) *be a variation of f*. *Then*

2.11 $$\delta (\sigma^{2})[f;\nu ]=-2e_{0}\delta e_{0}[f;\nu ]+\int_{0}^{\omega }x^{2}\nu (x)\;dx,\;\text{where}\;\delta e_{0}[f;\nu ]=\int_{0}^{\omega }x\nu (x)\;dx.$$

The first equation in (2.11) is the change in variance, to first order in *ɛ*, resulting from replacing the deaths distribution *f*(*x*) by *f*_*ɛ*_(*x*) = *f*(*x*) + *ɛν*(*x*) (i.e. if the deaths distribution is perturbed slightly by *ν*). Intuitively, then, the first equation in (2.11) tells us that when *e*_0_ is large, an increase in *e*_0_ is likely to result in a decrease in *σ*^2^, since in this case the first term in the equation will be a negative number likely larger in magnitude than the second term in the equation. (We say ‘likely’ because the actual value of *δ*(*σ*^2^)[*f*; *ν*] depends in a complex way on *ω*, *ν* and *e*_0_.) The interested reader is invited to consult (B 9) in appendix B and the calculations thereafter, where we illustrate theorem 2.5 for the theoretical example of a hyperbolic mortality case.

The results obtained thus far exhaust our theoretical study of variance curves. Altogether, they provide global insights into the shape of variance curves for terminal populations (satisfying assumptions (A 1) and (A 2)) and elucidate the precise conditions under which locally the curve increases, decreases or remains constant (theorem 2.4). In the next section, we begin the process of applying these results to real-world populations.

## 3. Variance Curve Hypotheses

While theorems 2.1–2.4 do not prescribe a specific shape for terminal populations’ variance curves, when considered in the context of what we know of real-world populations they do suggest a variety of properties of variance curves that we will below formulate as hypotheses and later explore empirically.

Our first hypothesis is inspired by the idealized variance curve in figure 1*a*. That variance curve has a unique absolute maximum and does not change direction before or after that maximum. We will refer to variance curves with this shape as ‘fundamental variance curves’.

**Definition 3.1.**

Let *P* be a terminal population with a continuous variance curve *G*(*γ*). We will call the variance curve a *fundamental variance curve of P* if *G*(*γ*) has a unique absolute maximum, increases before that maximum and decreases afterwards.

In reality, a real-world population’s variance curve is unlikely to match the idealized shape of a fundamental variance curve. But our first hypothesis posits that a terminal population’s variance curve ‘contains’ a fundamental variance curve in the following sense.

*The Variance Curve Pattern Hypothesis.* Let *P* be a terminal population whose variance curve *G*(*γ*) satisfies assumptions (A 1) and (A 2) from §2. Then there exists at least one *α*(*t*), where *G*(*α*) is a fundamental variance curve, such that *γ*(*t*) = *α*(*t*) + *ɛ*(*t*) and |*ɛ*(*t*)| ≪ max{*G*(*α*)} for all *t* in the domain of *γ*(*t*).

**Figure 2.**
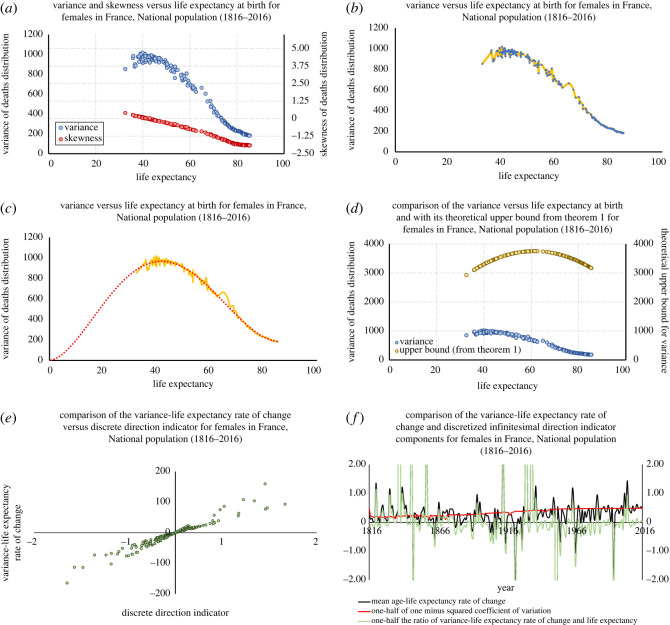
A plot of the skewness of the deaths distribution for females in France from 1816 to 2016 (panel (*a*), red, right axis), the variance of the deaths distribution (panels (*a*) and (*b*), blue, left axis), its smoothing (panels (*b*) and (*c*), gold), and a best-fit fifth-order polynomial approximation to the data (panel (*c*), in red). Panel (*d*) replaces the skewness data in panel (*a*) with the upper bound in (2.4) with *ω* = 122.5. Panel (*e*) plots the variance–life expectancy rate of change Δ*σ*^2^/Δ*e*_0_ against the discrete direction indicator *I* from (2.6) for 190 of the 200 adjacent life tables in the dataset; 10 extreme outliers we excluded for ease of visualization. Panel (*f*) shows the yearly changes in (1/2*e*_0_)(Δ*σ*^2^/Δ*e*_0_) (green), $\Delta \bar{x}/\Delta e_{0}$ (black) and $\frac{1}{2}(1-v^{2})$ (red).

This hypothesis posits, in other words, that it is always possible to decompose the variance curve *G*(*γ*) of a terminal population (satisfying assumptions (A 1) and (A 2)) into the sum of a fundamental variance curve *G*(*α*) and what amounts to a (small) perturbation driven by the function *ɛ*(*t*). Visually, it posits that a terminal population’s variance curve can always be seen as consisting of small oscillations about a fundamental variance curve. (The reader is invited to skip to figure 2*c* for an illustration of this.)

This first hypothesis still retains some interest in the *t*-parameter underlying variance curves. But, returning to the discussion in the previous section, let us now shift perspective to focus solely on the shape of a variance curve and, in particular, the variance–life expectancy correlations it may feature. This yields our second hypothesis.

*The Variance Curve Correlation Hypothesis.* Let *P* be a terminal population whose variance curve *G*(*γ*) satisfies assumptions (A 1) and (A 2) from §2. Then the correlation between life expectancy and the variance in *P*’s deaths distribution changes from positive to zero to negative as life expectancy increases from 0 to *ω*.

For example, the variance curve in figure 1*a* satisfies this hypothesis: *σ*^2^ and *e*_0_ are positively correlated for low life expectancies, uncorrelated in a neighbourhood of the maximum of *σ*^2^ and negatively correlated after that maximum.

Finally, let us now incorporate into our arguments what is known about *real-world* (terminal) populations’ mortality trajectories. Natural populations’ survival curves fall broadly into one of three types [20–22]: Type III curves with very high mortality at young ages (e.g. goldfish), Type II curves that have a constant proportion of individuals dying over time (e.g. turtles), and Type I curves characterized by low mortality levels until old age (e.g. modern humans). Now, because mortality in Type III and I populations is concentrated (in early and old ages, respectively), the associated variances in the death distributions of such populations will be low. By contrast, in Type II populations we expect high variances in the deaths distributions due to the near uniform spread in mortality across age. We will refer to this variance–type connection as the 'Variance Curve Type Hypothesis'.

*The Variance Curve Type Hypothesis.* Let *P* be a terminal population whose variance curve *G*(*γ*) satisfies assumptions (A 1) and (A 2) from §2. Then the variance in *P*’s deaths distribution is positively correlated with life expectancy in the population’s Type III states, uncorrelated in its Type II states and negatively correlated in its Type I states.

**Figure 3.**
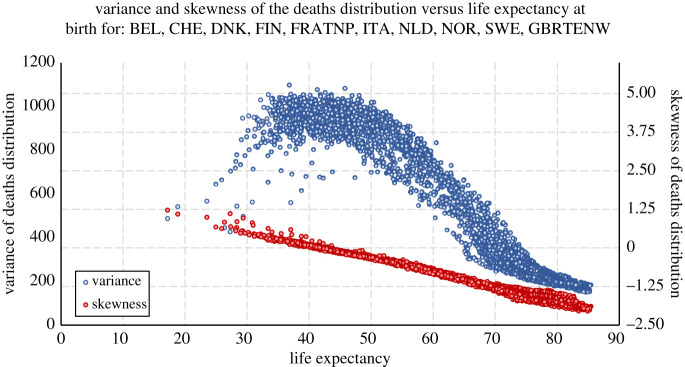
Plots of the variance in age at death (left axis, blue data) and skewness of the deaths distribution (right axis, red data) versus life expectancy for females and males in the 10 countries in the Human Mortality Database [23] with the oldest data (3343 yearly life tables for males and females spanning 1751–2016; see table 2 in appendix D for more information): Belgium (BEL), Siwtzerland (CHE), Denmark (DNK), Finland (FIN), France (total population; FRATNP), Italy (ITA), The Netherlands (NLD), Norway (NOR), Sweden (SWE) and the UK (England and Wales, total population; GBRTENW).

This hypothesis posits that the population’s survival curve types are sufficient to explain the correlation phases (positive, zero, negative) between life expectancy and the variance of ages at death in the population. Verifying this hypothesis in practice—which we will do in §4—requires quantifying type. In [22] (see, specifically, fig. 3 therein and the discussion surrounding it), it was suggested that type could be quantified via the skewness of the underlying deaths distribution, such that a positively skewed deaths distribution is indicative of a Type III state, near zero skewness a Type II state and negative skewness a Type I state. We have not found a better type metric advocated in the literature so we will use this skewness metric herein. However, we note here upfront the imperfect nature of this type indicator: since in practice survival curve types are identified *qualitatively*, any *quantitative* metric of type—including the skewness of the deaths distribution—will ultimately be an imperfect type identifier. With this choice of type indicator and keeping this caution in mind, we can rephrase the Variance Curve Type Hypothesis above as follows (we have appended ‘Quantified’ to the title to remind the reader of the above discussion).

*The Quantified Variance Curve Type Hypothesis.* Let *P* be a terminal population whose variance curve *G*(*γ*) satisfies assumptions (A 1) and (A 2) from §2. Then the variance in *P*’s deaths distribution is positively correlated with life expectancy when the skewness of population’s deaths distribution is approximately positive, uncorrelated when that skewness is approximately zero and negatively correlated when that skewness is approximately negative.

For interested readers, we illustrate these hypotheses in appendix B, §B.1 using the hyberbolic mortality model theoretical example. In the next section, we examine the empirical evidence in humans for the Pattern, Correlation and Quantified Type Hypotheses.

## 4. Empirical support for the Variance Curve Hypotheses

We herein consider only human populations, since these are the populations we have the most reliable data for. We first illustrate our results and hypotheses in the French female population between 1816 and 2016, and then turn to a broader study of the 10 countries in the Human Mortality Database (HMD) [23] with the oldest data.

Our first step is to verify the various hypotheses we have made in §2. The easiest of these to verify are hypotheses (A 1) and (A 2). Like all real-world populations, human populations display a variance in their ages of death; thus hypothesis (A 1) is satisfied. Hypothesis (A 2), as well as any continuity or smoothness hypotheses made of life table functions (e.g. the deaths distribution), can be satisfied by construction. Specifically, since demographic data—like those contained in the life tables that make up the HMD—are discrete, one can interpolate those data (including the implied variance curve data) with functions satisfying the hypotheses made in §2. (We illustrate this explicitly in figure 2*b*.) Thus hypotheses (A 1) and (A 2) and any associated continuity or smoothness assumptions on the relevant life table functions either hold manifestly in real-world populations or can be made to hold by appropriately constructing the continuous versions of the relevant life table functions. The hypothesis that *ω* is finite, however, is different. This question has been studied extensively for human populations (see [27] and references therein), and there appears to be no scientific consensus as yet. Some research using statistical methods indicates that *ω* is indeed finite for humans (see [28] and references therein). But other research concludes that the ‘data does not support that there is a finite upper limit to the human lifespan’ [29]. This state of affairs makes clear that *ω* = finite and *ω* = ∞ are evidently both defensible assumptions in the literature, and thus we will proceed as though the former is the true statement (that *ω* is finite). (In addition, we point out that this assumption is the only empirically tractable hypothesis—one cannot verify *empirically* that a population’s maximum lifespan is infinite.)

Having now argued that the hypotheses we made in §2 are satisfied by human populations, we now proceed to investigate the results developed in §§2 and 3.

### 4.1. The variance curve for French females, 1816–2016

We extracted yearly life table data for this population from the HMD [23] (201 yearly life tables, 1816–2016) and calculated the relevant quantities associated with the deaths distributions needed for our analyses. Let us now describe how the results support the three hypotheses from the previous section and illustrate our earlier theoretical results.

#### 4.1.1. The Variance Curve Correlation and Quantified Type Hypotheses

Figure 2*a* plots the variance (blue, left axis) and skewness (red, right axis) of the population’s deaths distributions as a function of the life expectancy at birth. We observe that the data conforms to the Variance Curve Correlation Hypothesis: the variance–life expectancy correlation changes from positive to zero to negative as life expectancy increases from its smallest value in the dataset. To evaluate the Quantified Variance Curve Hypothesis, let us return to figure 2*a*. Looking now at the skewness data therein, we see that the population’s deaths distributions were positively skewed for $e_{0}⪅40$, precisely the region over which the variance–life expectancy correlation is positive; had near zero skewness for *e*_0_ ≈ 40, where the variance and life expectancy are uncorrelated; and were negatively skewed for *e*_0_ > 40, where the variance–life expectancy correlation is negative. This conforms to our Quantified Variance Curve Type Hypothesis.

#### 4.1.2. The Variance Curve Pattern Hypothesis

The golden curve in figure 2*b* is an interpolation of the (blue) data from panel (*a*); it is an approximation to the ‘true’ variance curve (the curve one would obtain from life tables created more frequently than at yearly intervals). The red curve in panel (*c*) is a best-fit fifth-order polynomial regression. (This is the smallest-order polynomial consistent with the bounds in (2.4).) As panel (*c*) shows, the approximation to the ‘true’ variance curve can be seen as the sum of a ‘fundamental variance curve’ (the red curve in the figure) and a small perturbation to that curve. This conforms to our Variance Curve Pattern Hypothesis.

Finally, let us illustrate theorems 2.1 and 2.4. Figure 2*d* plots the upper bound in (2.4)—using *ω* = 122.5, roughly the oldest known person to have lived (French female Jeanne Calment [30])—for each *e*_0_-value in the dataset (yellow) along with the associated *σ*^2^-values (blue). We see clearly that the variance values respect the upper bound. To illustrate theorem 2.4, given that the largest variance value (*σ*^2^ ≈ 1, 019.4) in the dataset occurred in 1824—this is the maximum value of the (blue) data plotted in figure 2*a*—let us illustrate the theorem by comparing the mortality profiles of 1822, 1824 and 1827. In table 1 we provide the summary statistics (calculated directly from the associated life tables) and calculate the quantities from theorem 2.4 needed for our analysis.

**Table 1.** Summary of mortality and survival data for French females, 1822, 1824 and 1827; rates of change of the variance in age at death, discrete direction indicator for French females: 1822 compared with 1824, and 1824 compared with 1827.

year	*e*_0_	*σ*^2^	$\bar{x}$	comparison	Δ*σ*^2^/Δ*e*_0_	*I* (2.6)
1822	39.04	1008.37	32.43			
1824	39.97	1019.39	32.74	1822 and 1824	11.83	0.1660
1827	40.14	973.37	32.19	1824 and 1827	−270.70	−3.3699

We note that life expectancy increased in both comparisons, but the variance in age at death increased in the first (the 1822 and 1824 comparison) and decreased in the second (the 1824 and 1827 comparison). These different outcomes were correctly predicted by *I*, the discrete direction indicator from (2.6), which indicated the correct direction of effects on the variance as a result of the increases in life expectancy. To further illustrate theorem 2.4, we ran similar analyses comparing adjacent years (e.g. 1824 to 1825) for all 201 life tables in the dataset. In figure 2*e*, we plot the associated rates of change Δ*σ*^2^/Δ*e*_0_ and their corresponding discrete direction indicator values (equation (2.6)); about 5% of the data (10 life tables) are not shown due to being extreme outliers. As the figure suggests, and as we verified in *all* 200 adjacent life table comparisons, the discrete direction indicator *I* correctly predicts the sign of the variance rate of change Δ*σ*^2^/Δ*e*_0_ and thus the increase or decrease of the variance in age at death due to a change in life expectancy. As for the infinitesimal direction indicator (2.7), we note that it should only be used when one has an analytical expression relating *σ*^2^ to *e*_0_ (as in the case of the hyperbolic mortality model illustrated in appendix B). However, because when discretized it yields the first two terms on the right-hand side of (2.6), we can use this discretization to explore how well using just those first two terms of *I* predicts the correct sign for $\frac{\Delta \sigma^{2}}{\Delta e_{0}}$. Surprisingly, in 196 of the 200 comparisons that truncation of *I* predicted the correct sign. Figure 2*f* visualizes this by plotting $\frac{1}{2}(1-v^{2})$ (red), $\Delta \bar{x}/\Delta e_{0}$ (black), and (1/2*e*_0_)(Δ*σ*^2^/Δ*e*_0_) (green) for the dataset. When the black curve is above (below) the red curve, then the aforementioned discretization of the infinitesimal indicator (2.7) is positive (negative), and hence we would expect the green curve—the values of (1/2*e*_0_)(Δ*σ*^2^/Δ*e*_0_)—to be above (below) the *x*-axis. We observe that positive values for the green curve were more common pre-1870 than post-1870. This is because after the 1870s the rate of change in the mean age with respect to life expectancy (the black curve in figure 2*f*) was smaller than the increasingly higher levels of $\frac{1}{2}(1-v^{2})$ (the red curve in the figure), which themselves were driven by the general downward trend in the variance of age at death (figure 2*a*) in that period. This downward trend for the variance may be reversed in the future, however, if the rate of change of the mean age with respect to life expectancy in the population exceeds the prevailing levels of (1/2)(1 − *v*^2^). (Indeed, we observe some limited evidence of this in the last few years in the dataset, as indicated by the positive values of the green curve in figure 2*f ca* 2016.)

### 4.2. Variance versus life expectancy for the 10 countries in the human mortality database with the oldest data

We now broaden our dataset to the 10 countries in the Human Mortality Database [23] with the oldest data to show that the regularities just described in the French data also extend to these other countries. Figure 3 plots *σ*^2^ versus *e*_0_ for those 10 countries in the dataset (3343 yearly life tables for males and females spanning 1751–2016): Belgium (BEL), Switzerland (CHE), Denmark (DNK), Finland (FIN), France (total population; FRATNP), Italy (ITA), The Netherlands (NLD), Norway (NOR), Sweden (SWE) and the UK (England and Wales, total population; GBRTENW); see table 2 in appendix D for additional details. We again see an overall pattern similar to the data for French females from figure 2*a*—the variance in the age at death is overall positively correlated with life expectancy for $e_{0}⪅40$, uncorrelated for *e*_0_ ≈ 40, and negatively correlated for *e*_0_ > 40. This conforms to the Variance Curve Correlation Hypothesis. We also again see that the variance is positively correlated with life expectancy for $e_{0}⪅40$, where the skewness of the deaths distribution is positive (indicating a Type III state in the population); uncorrelated for *e*_0_ ≈ 40, where the skewness indicates a Type II state; and negatively correlated for *e*_0_ > 40, where the skewness indicates a Type I state. This conforms to our Quantified Variance Curve Type Hypothesis. These conclusions also hold for the individual countries’ variance curves as demonstrated in figure 7 (in appendix D).

**Figure 4.**
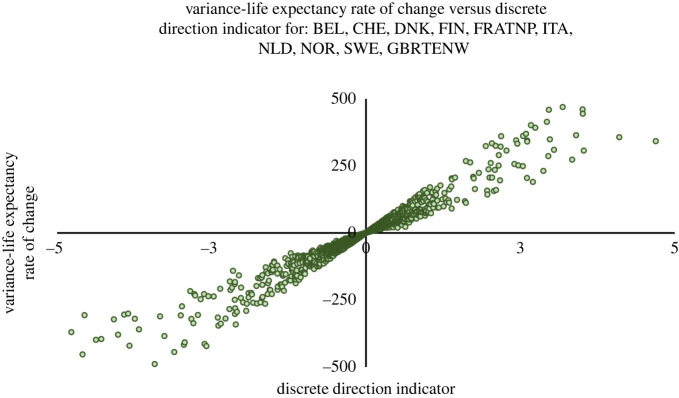
A plot of the variance–life expectancy rate of change Δ*σ*^2^/Δ*e*_0_ against the discrete direction indicator*I*from (2.6) for females and males in the 10 countries in the Human Mortality Database [23] with the oldest data (3343 yearly life tables for males and females spanning 1751–2016; see table 2 in appendix D for more information): Belgium (BEL), Switzerland (CHE), Denmark (DNK), Finland (FIN), France (total population; FRATNP), Italy (ITA), The Netherlands (NLD), Norway (NOR), Sweden (SWE) and the UK (England and Wales, total population; GBRTENW); 212 extreme outliers were excluded for ease of visualization.

We also repeated our investigation of theorem 2.4 for this new dataset. We again calculated Δ*σ*^2^/Δ*e*_0_ and *I* for all adjacent life tables in the dataset. In figure 4, we plot all but about 6% (roughly 200) of these pairs, excluding those extreme outliers for ease of visualization. As the figure suggests, and as we verified, *I* correctly predicts the direction of change in *σ*^2^ in *all* the adjacent life tables in the dataset. We also constructed the same discretization of *J* described earlier and calculated it for all the life tables. We found that it correctly predicted the direction of the change in variance in over 98% of the comparisons. We also generated analogues of figure 2*f* for each country to document the particular dynamics between the variance in age at death, mean age and the coefficient of variation in each country. These figures are included in figures 8–17 in appendix D; they are divided into male and female plots for four periods—pre-1900, 1900–1949, 1950–1999 and 2000–2016—for ease of visualization.

Finally, we calculated the bound (2.4) from theorem 2.1 for this broader dataset and once again found the data to be in accordance with it; figure 18 in appendix D illustrates this.

## 5. Conclusion

Variation is essential to evolution, and over a small range of life expectancies it is reasonable to expect that species’ varying lifestyles coupled with evolutionary forces should result in a diversity of variance curve shapes. Numerous empirical studies (referenced in the Introduction) have documented empirical regularities between the uncertainty in the age at death of real-world populations and their life expectancies, leading in particular to the correlations between $e^{†}$ and *e*_0_, and *σ*^2^ and *e*_0_ cited in the Introduction. However, the theoretical explanations for these empirical correlations have yet to be elucidated. Our work contributes to this research in three central ways: by establishing three theoretical results (theorems 2.1–2.5), three hypotheses and several new empirical regularities.

The first theorem—theorem 2.1—is, in our opinion, the more foundational one, for it places an upper bound on the variance in the deaths distribution associated with a given life expectancy for terminal populations. In particular, it forces these populations’ variance values to decrease toward zero as their life expectancies approach 0 or *ω*. This necessarily implies that the variance curve of a terminal population must have a maximum value. Theorems 2.4 and 2.5 then quantify how the variance in age at death changes on its way to or from that maximum value in response to an underlying change in life expectancy, and provide explicit conditions which predict when the variance will increase or decrease. These conditions depend on the sign of the direction indicators *I* and *J*, and as we illustrated in the discussion after theorem 2.4, those signs depend on a particular interplay of demographically meaningful attributes in the population (e.g. whether $d\bar{x}/de_{0}>(1-v^{2})/2$). These results provide, therefore, a direct link between a population’s demographic state—measured by parameters like *v* and $\bar{x}$—and its variance trajectory along its variance curve.

Our second central contribution is the formulation of our three hypotheses. Each hypothesis formalizes the properties of variance curves’ shapes and makes new connections in ways that can ground future research and catalyse future discoveries. For example, our Type and Quantified Type Hypotheses furnish new connections between survival curve types, the skewness of the deaths distribution, and the three-phased variance–life expectancy correlation pattern documented in modern human populations. As such, these hypotheses pave the way for investigating the broader role that the skewness of the deaths distribution—or survival curve type—plays in driving lifespan inequality–life expectancy interrelationships.

Our third contribution is the new empirical regularities documented in the empirical studies we presented in §4. Those studies not only support all our hypotheses in modern human populations, they also showcase the direction indicators (2.6) and (2.7) and their usage for accurately predicting how the variance in age at death changes in response to changes in life expectancy. In particular, these results and analyses make clear that when life expectancy changes, the impact on the variance in age at death (increase, decrease or unchanged) is determined—with high accuracy—by comparing the rate of change of the mean age in the stationary population with respect to life expectancy, and the quantity (1/2)(1 − *v*^2^) (as theorem 2.4 showed). This is a new empirical regularity. Our empirical studies also present strong evidence for the capability of the skewness of the deaths distribution to distinguish between the three correlation phases we have been concerned with in this article, another new regularity. We stress that these new regularities stemmed directly from the theoretical study of variance curves we undertook in this article. In that direction, we now discuss one ramification and two potential extensions of our results.

**Figure 5.**
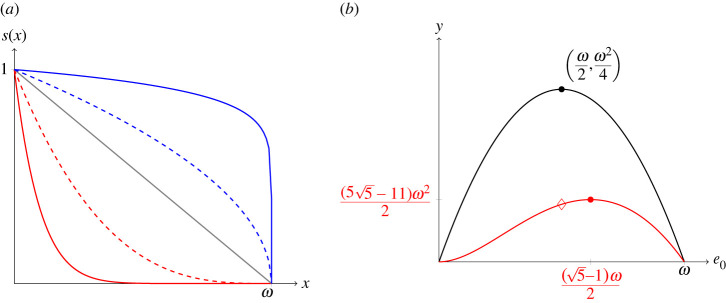
(*a*) The graph of *s*(*x*) = (1 − *x*/*ω*)^*a*^ for *a* = 10 (solid red), *a* = 3 (dashed red), *a* = 1 (grey), *a* = 0.5 (dashed blue), and *a* = 0.1 (solid blue) associated with the hyperbolic mortality function (B 1). (*b*) The graph of the upper bound in (2.4), *y* = *ψ*(*e*_0_) = *ω*^2^/4 − (*e*_0_ − *ω*/2)^2^ (in black), along with the function *y* = *σ*^2^(*e*_0_) from (B 3) (in red). The two points indicated are the maxima of the respective functions. The diamond on the red curve is the point on the curve at which *e*_0_ = *ω*/2 (which corresponds to *a* = 1 according to (B 3)).

**Figure 6.**
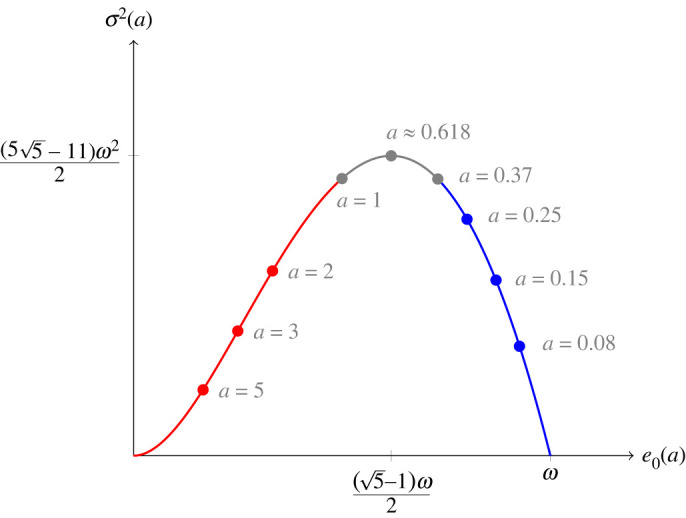
The parametric plot (*e*_0_(*a*), *σ*^2^(*a*)), using (B 3), for *a* > 0, with selected *a*-values overlayed in grey. All points on the red-coloured portion of the curve have *a* < 1, all on the blue-coloured portion have *a* < 0.37, and all on the grey-coloured portion have 0.37 ≤ *a* ≤ 1.

**Figure 7.**
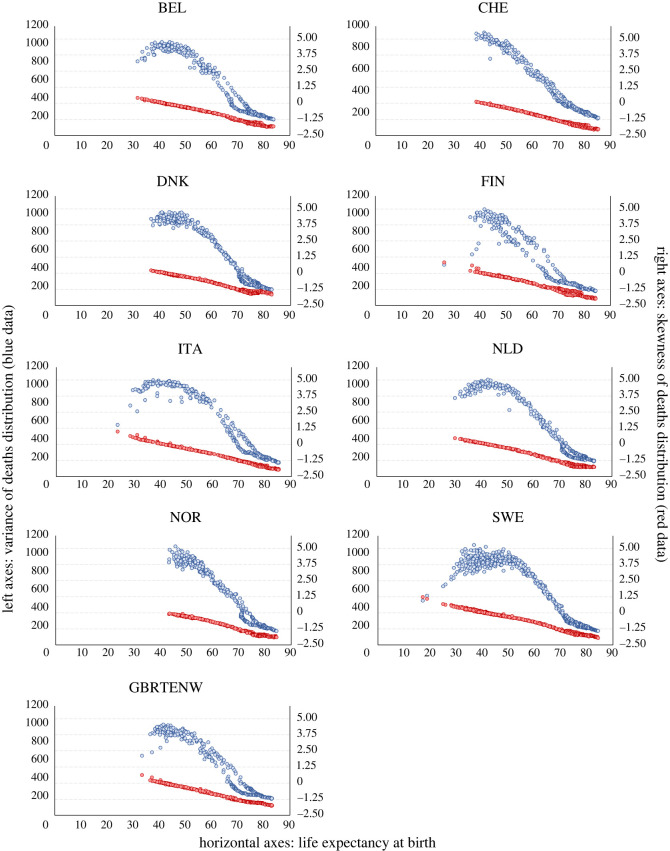
Plots of the variance in age at death (left axes, blue data) and skewness of the deaths distribution (right axes, red data) versus life expectancy for females and males in the following Human Mortality Database [23] countries (see table 2 in appendix D for more information): Belgium (BEL), Switzerland (CHE), Denmark (DNK), Finland (FIN), Italy (ITA), The Netherlands (NLD), Norway (NOR), Sweden (SWE) and the UK (England and Wales, total population; GBRTENW).

**Figure 8.**
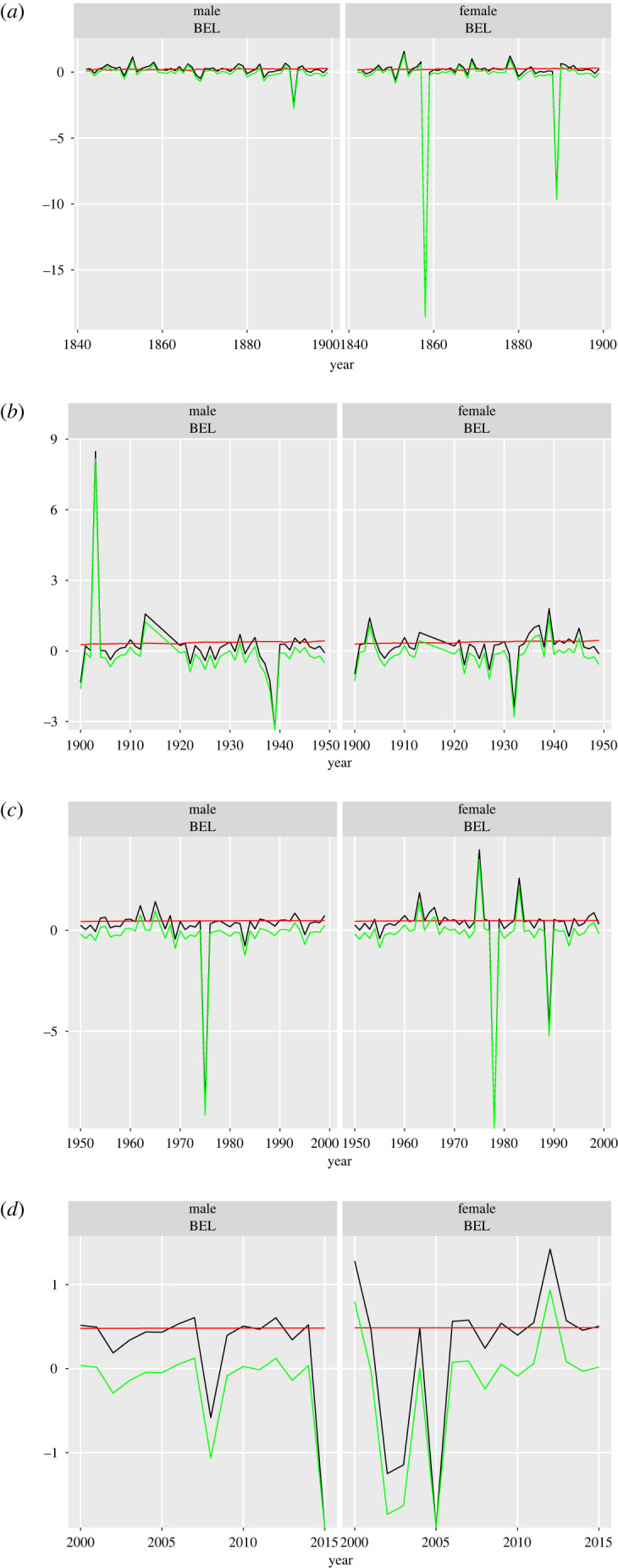
Plots of the yearly changes in (1/2*e*_0_)(Δ*σ*^2^/Δ*e*_0_) (green), $\Delta \bar{x}/\Delta e_{0}$ (black) and (1/2)(1 − *v*^2^) (red) for BEL (169 total yearly life tables from the HMD [23]).

**Figure 9.**
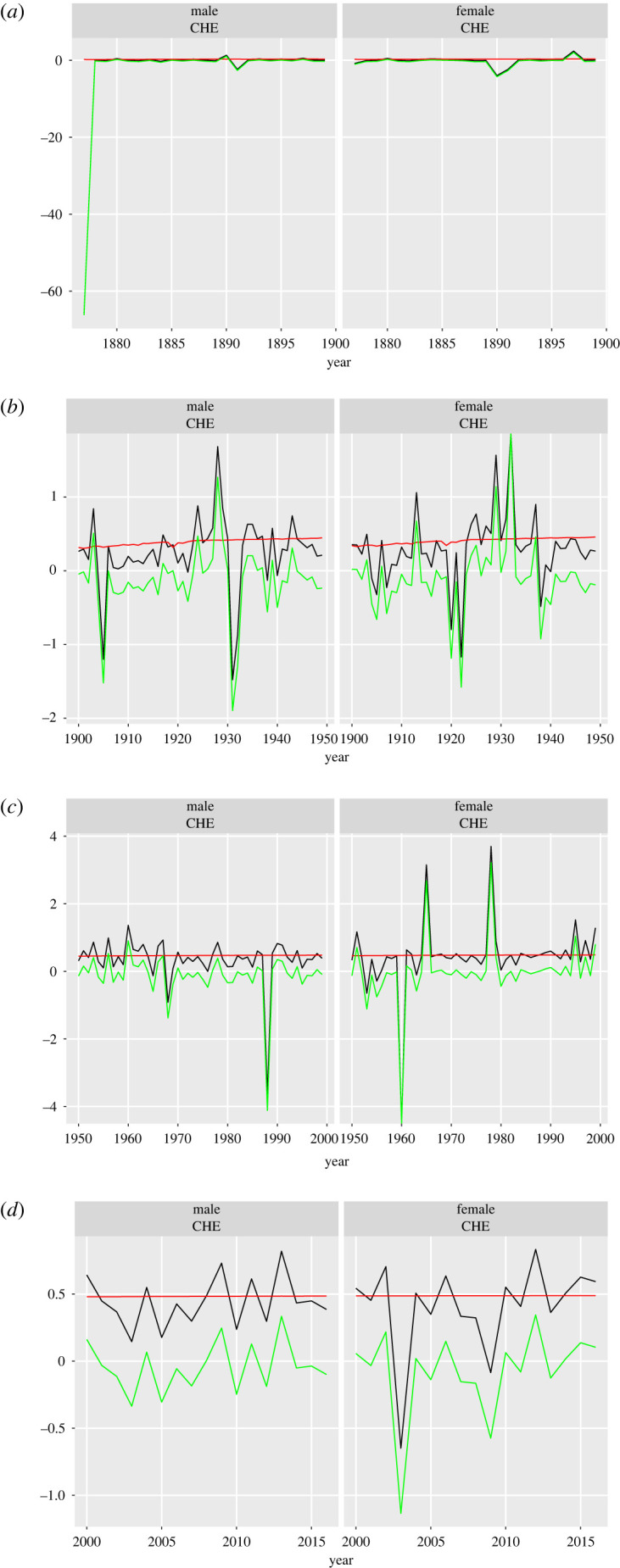
Plots of the yearly changes in (1/2*e*_0_)(Δ*σ*^2^/Δ*e*_0_) (green), $\Delta \bar{x}/\Delta e_{0}$ (black) and (1/2)(1 − *v*^2^) (red) for CHE (280 total yearly life tables from the HMD [23]).

**Figure 10.**
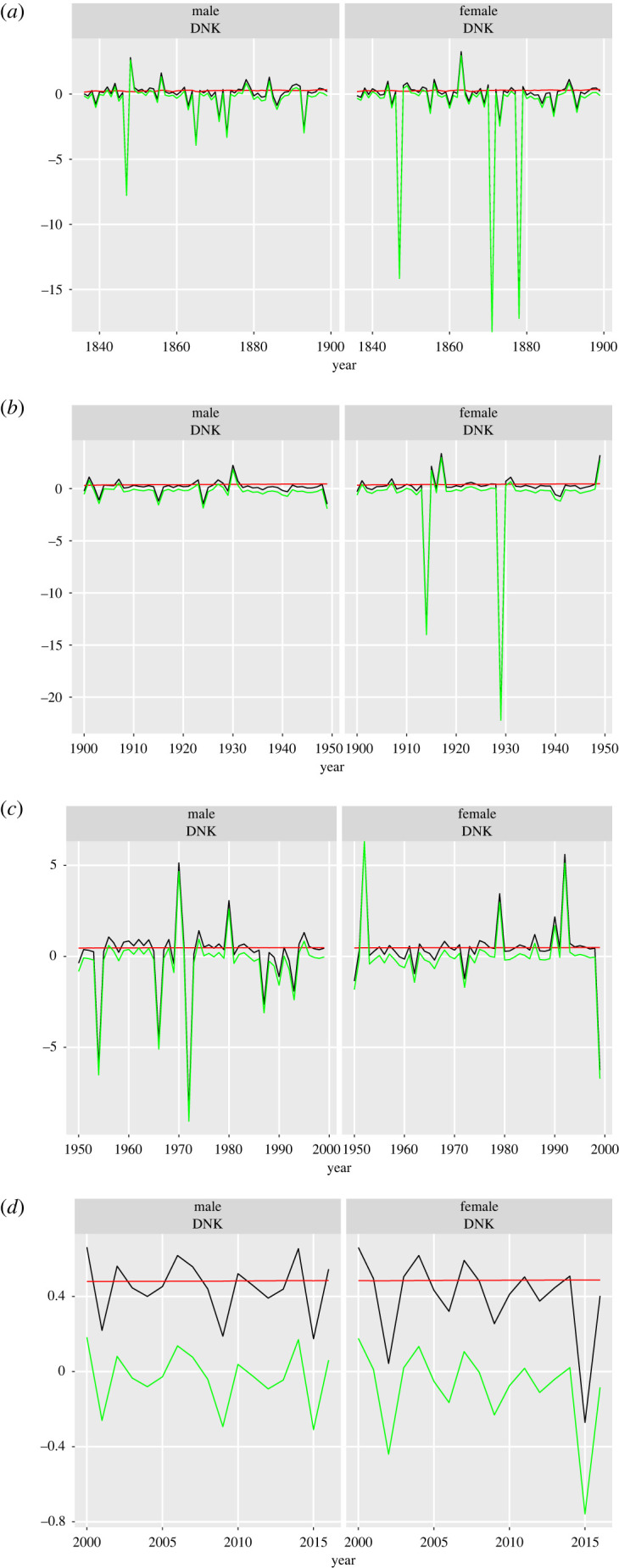
Plots of the yearly changes in (1/2*e*_0_)(Δ*σ*^2^/Δ*e*_0_) (green), $\Delta \bar{x}/\Delta e_{0}$ (black) and $\frac{1}{2}(1-v^{2})$ (red) for DNK (364 total yearly life tables from the HMD [23]).

**Figure 11.**
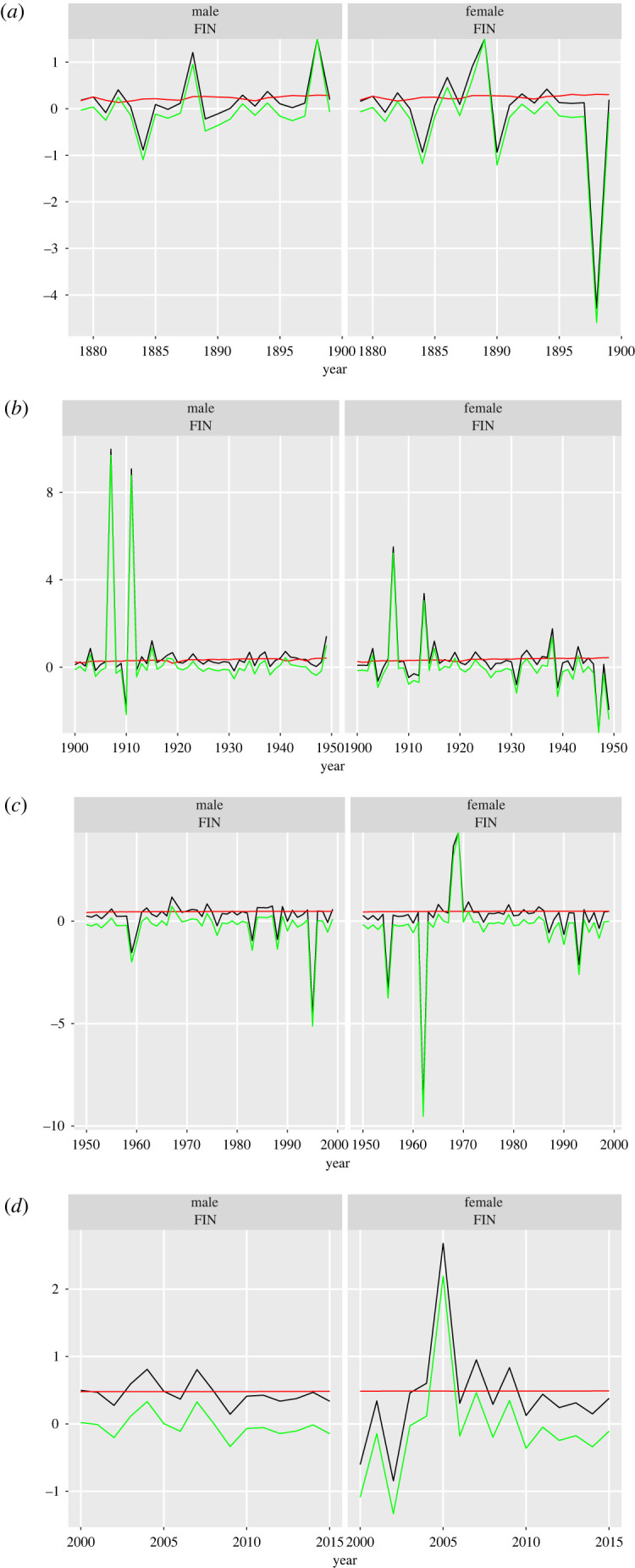
Plots of the yearly changes in (1/2*e*_0_)(Δ*σ*^2^/Δ*e*_0_) (green), $\Delta \bar{x}/\Delta e_{0}$ (black) and (1/2)(1 − *v*^2^) (red) for FIN (278 total yearly life tables from the HMD [23]).

**Figure 12.**
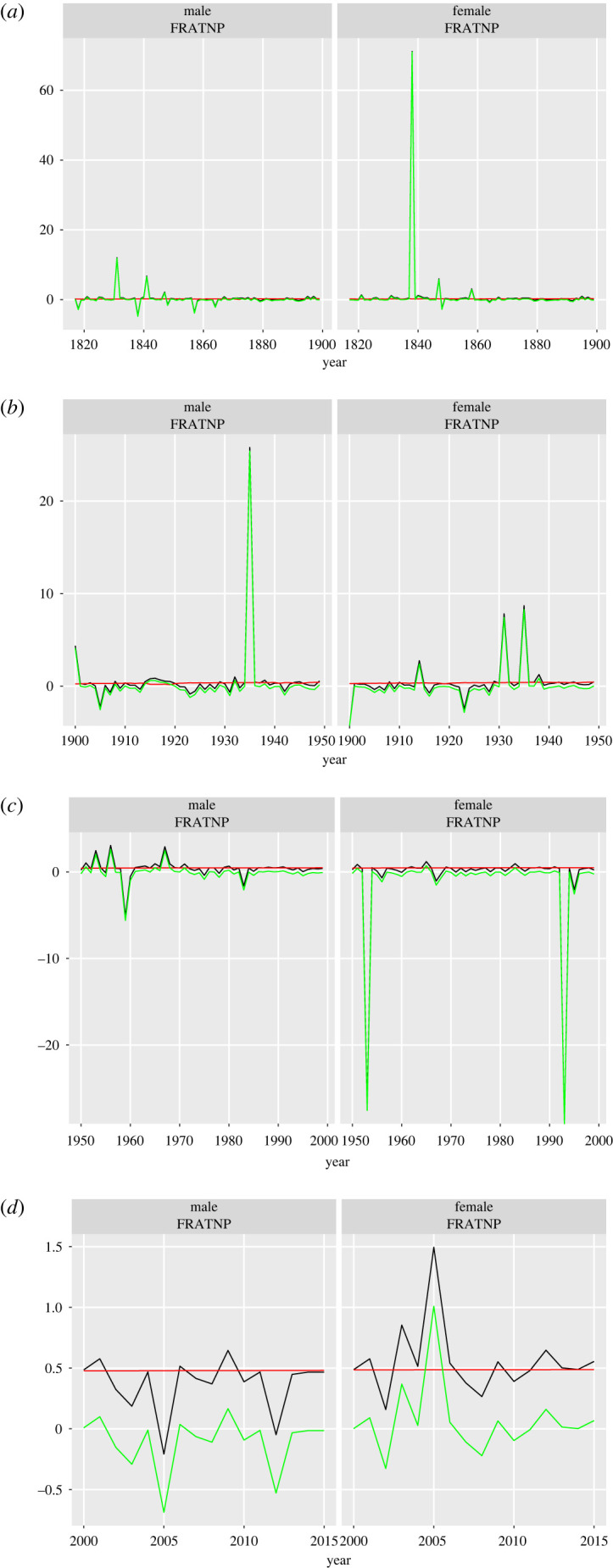
Plots of the yearly changes in (1/2*e*_0_)(Δ*σ*^2^/Δ*e*_0_) (green), $\Delta \bar{x}/\Delta e_{0}$ (black) and (1/2)(1 − *v*^2^) (red) for FRATNP (402 total yearly life tables from the HMD [23]).

**Figure 13.**
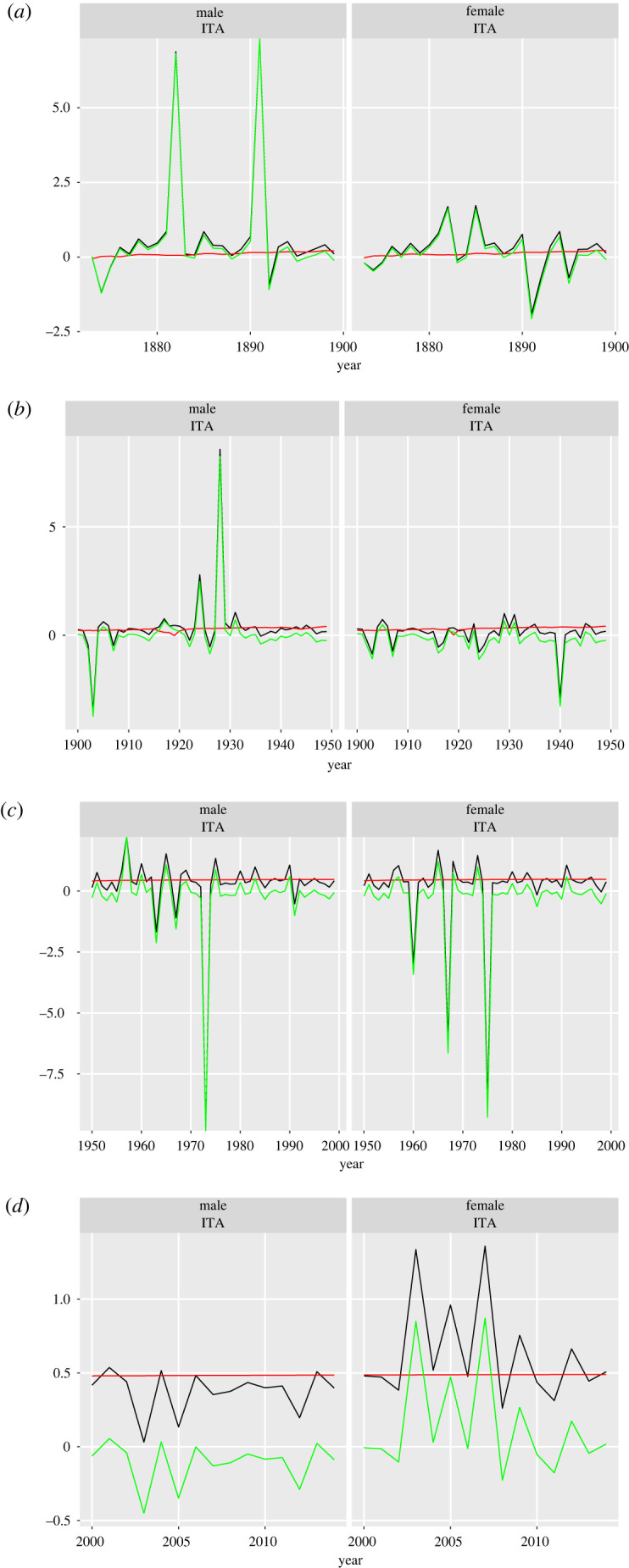
Plots of the yearly changes in (1/2*e*_0_)(Δ*σ*^2^/Δ*e*_0_) (green), $\Delta \bar{x}/\Delta e_{0}$ (black) and (1/2)(1 − *v*^2^) (red) for ITA (290 total yearly life tables from the HMD [23]).

**Figure 14.**
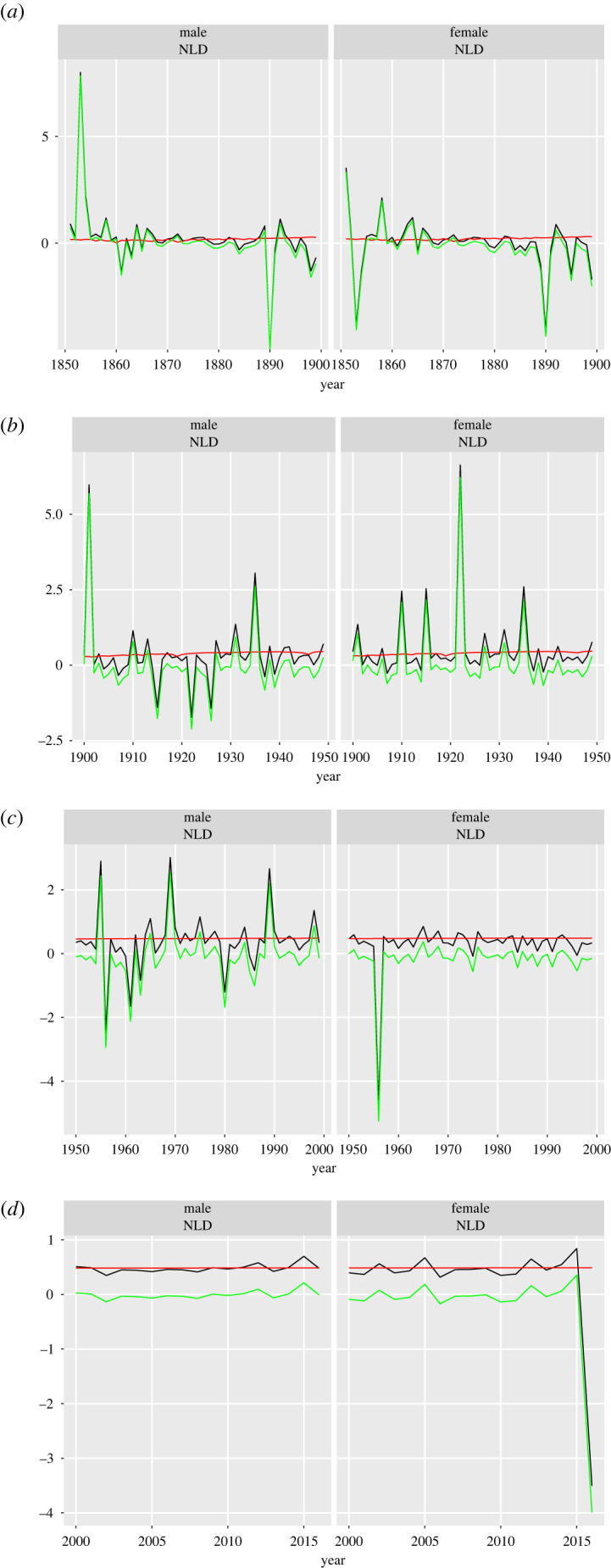
Plots of the yearly changes in (1/2*e*_0_)(Δ*σ*^2^/Δ*e*_0_) (green), $\Delta \bar{x}/\Delta e_{0}$ (black) and (1/2)(1 − *v*^2^) (red) for NLD (334 total yearly life tables from the HMD [23]).

**Figure 15.**
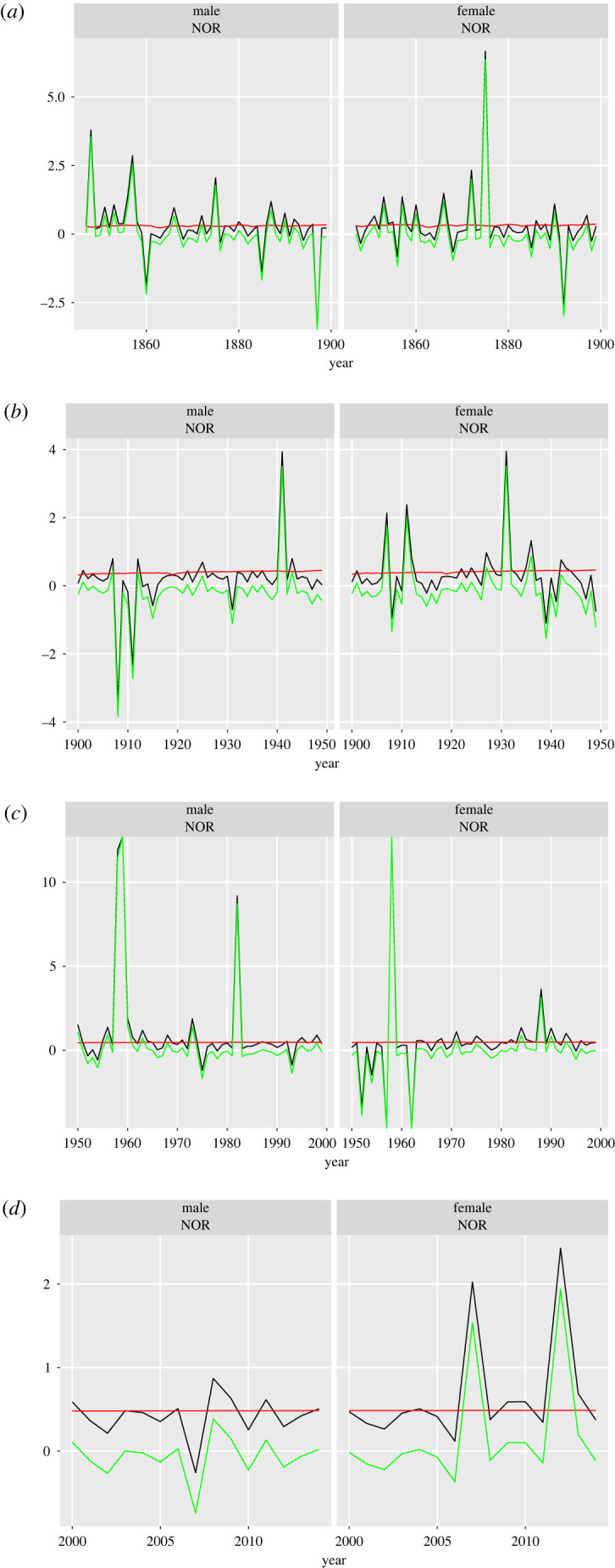
Plots of the yearly changes in (1/2*e*_0_)(Δ*σ*^2^/Δ*e*_0_) (green), $\Delta \bar{x}/\Delta e_{0}$ (black) and (1/2)(1 − *v*^2^) (red) for NOR (342 total yearly life tables from the HMD [23]).

**Figure 16.**
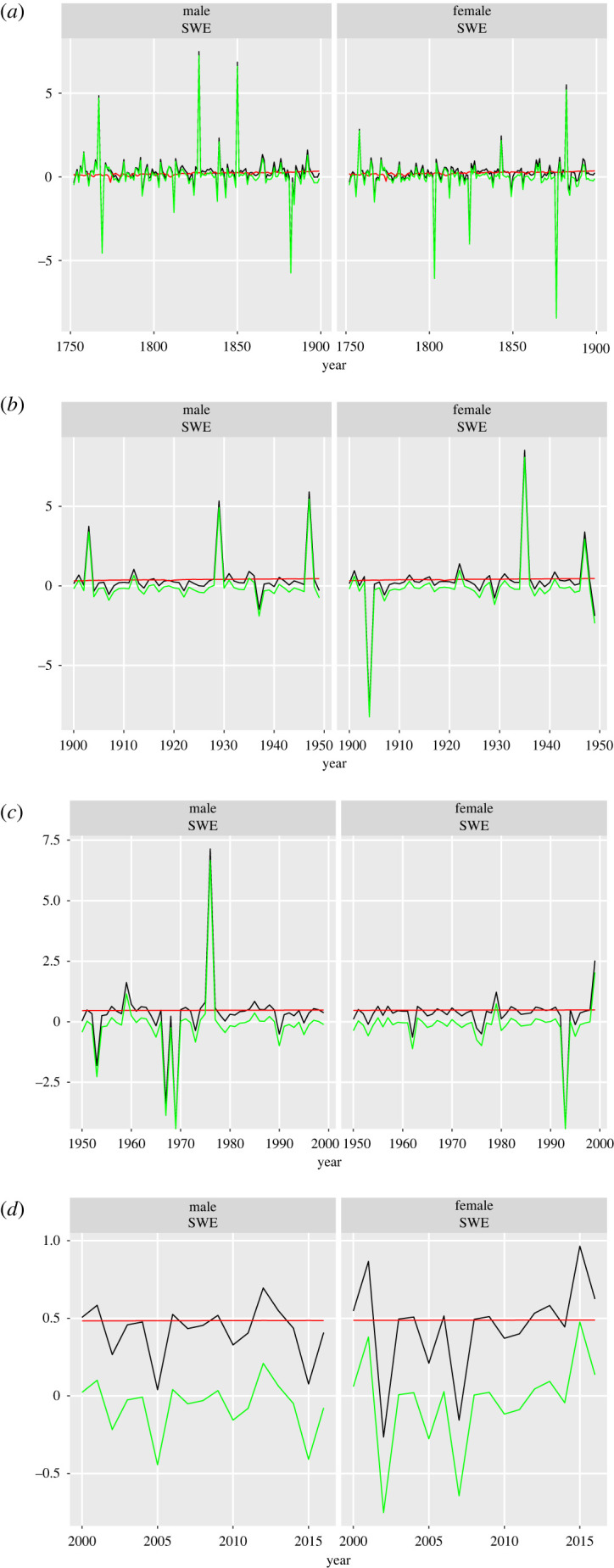
Plots of the yearly changes in (1/2*e*_0_)(Δ*σ*^2^/Δ*e*_0_) (green), $\Delta \bar{x}/\Delta e_{0}$ (black) and (1/2)(1 − *v*^2^) (red) for SWE (532 total yearly life tables from the HMD [23]).

**Figure 17.**
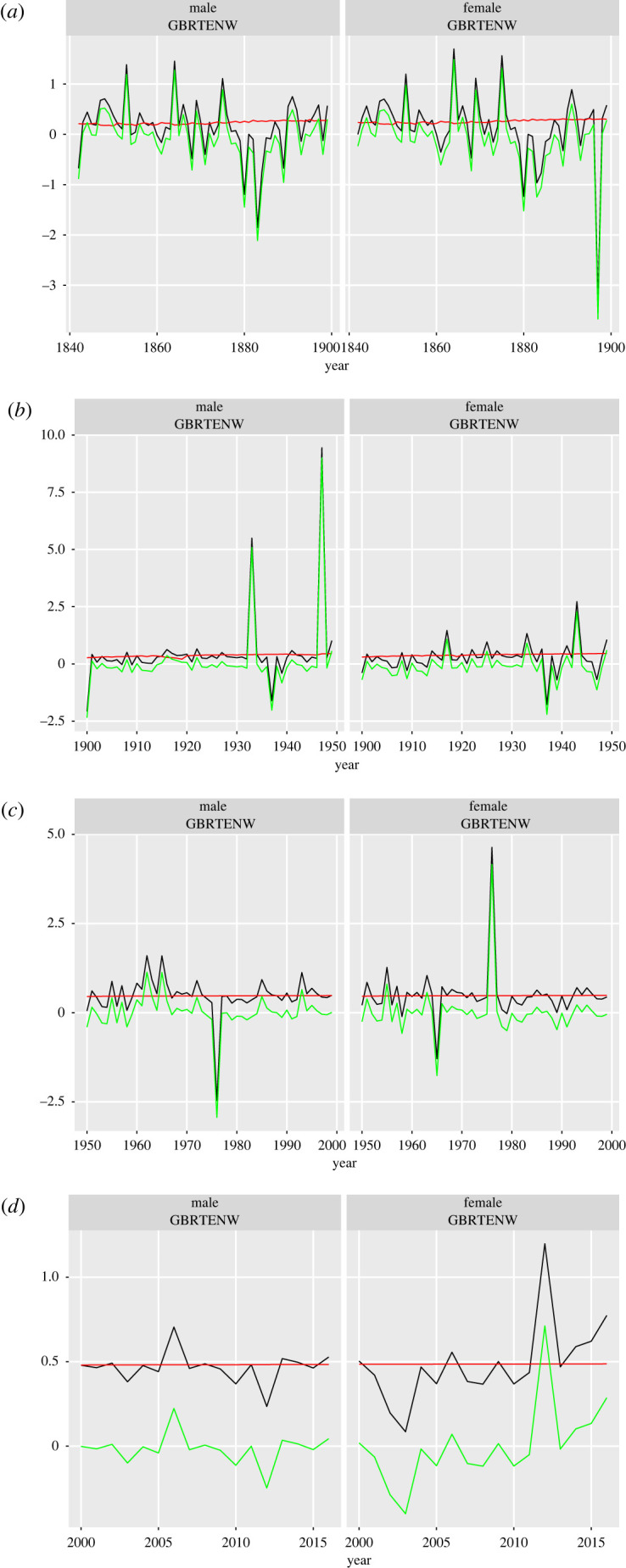
Plots of the yearly changes in (1/2*e*_0_)(Δ*σ*^2^/Δ*e*_0_) (green), $\Delta \bar{x}/\Delta e_{0}$ (black) and (1/2)(1 − *v*^2^) (red) for GBRTENW (352 total yearly life tables from the HMD [23]).

**Figure 18.**
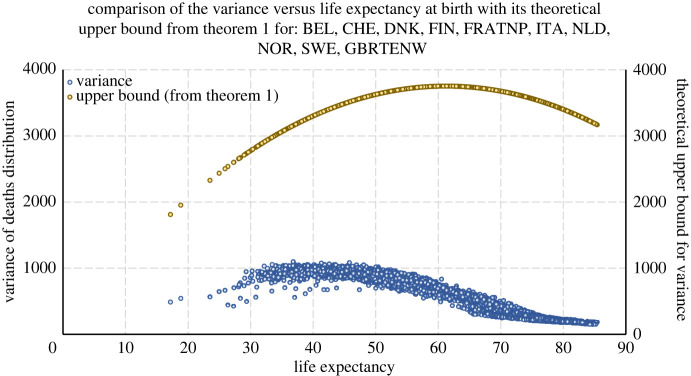
A plot of the variance in the age at death (blue) and the upper bound in that variance from theorem 2.1 (yellow) versus life expectancy for females and males in the 10 countries in the Human Mortality Database [23] with the oldest data (3343 yearly life tables for males and females spanning 1751–2016; see table 2 in appendix D for more information): Belgium (BEL), Switzerland (CHE), Denmark (DNK), Finland (FIN), France (total population; FRATNP), Italy (ITA), The Netherlands (NLD), Norway (NOR), Sweden (SWE) and the UK (England and Wales, total population; GBRTENW).

The ramification concerns the findings of [31], which documents evidence that in human populations the ‘average length of life is long and relative variation in lifespans is low’. The authors termed populations with this property ‘longevous populations’ and classified human populations as longevous populations. However, as already indicated by past empirical studies [12,14–16], and as confirmed in the data we analysed (figures 2–7), for $e_{0}\approx 40\;\mathrm{years}$ the variance in lifespan is at its maximum; thus modern human populations only became longevous when life expectancy began exceeding $e_{0}\approx 40\;\mathrm{years}$. For $e_{0}⪅40\;\mathrm{years}$, human populations were evidently in a ‘non-longevous’ state, which we might define as characterized by an ‘average length of life that is *short* and a relative variation in lifespans that is *high*’). Our theoretical results herein now provide the first theoretical explanation for why human variance curves must have *both* of these states: longevous and non-longevous. Indeed, so long as one assumes what we have assumed herein—including, for instance, that the maximum human lifespan is finite—then by our arguments following theorem 2.1, perpetually longevous or perpetually non-longevous states cannot occur. (This same finding is implicit in proposition 1 in [11] in the setting where lifespan inequality is measured via $e^{†}$.) Our work, therefore, contextualizes the findings of [31], provides a theoretical explanation for the findings of [12,14–16], and unifies these results into a broader three-phased pattern of change for the correlation between the variance in lifespan and life expectancy (figures 3–7) described by the Variance Curve Correlation Hypothesis. We therefore suggest that the Variance Curve Correlation Hypothesis is more characteristic of life expectancy–lifespan inequality relationships in human populations and provides a more nuanced understanding of how and when such relationships manifest as negative, zero or positive correlations than the longevous characterization. (In addition, we now have demographically meaningful indicators (*I* and *J* from (2.6) and (2.7), respectively) that tell us precisely when the variance in age at death increases or decreases when life expectancy changes, results that once again describe in detail how and when said variance changes.) An interesting direction for future work would be to investigate whether our theoretical results herein generalize to other lifespan inequality metrics—do our theorems hold, for example, with *σ*^2^ replaced by $e^{†}$, or by the ‘life table entropy’ $H=e^{†}/e_{0}$? In the latter case, an answer would provide a theoretical foundation for the empirical correlations documented elsewhere [6,32,33]. In this direction we are encouraged by the work of Permanyer & Shi [19], which derives bounds similar to that of (2.4) for other lifespan inequality metrics. Perhaps by following the same lines of reasoning in this article but starting with the bounds in [19] one may be able to derive results and insights similar to the ones presented herein.

Regarding the extensions of our results, the first natural extension is to the setting of truncated deaths distributions. Rather than working with the full deaths distribution *f*(*x*) (i.e. from birth), one could instead consider the ‘*a*-truncated deaths distribution’ *f*_*a*_(*x*) [17], the deaths distribution restricted to ages *x* ≥ *a*. Denoting by *σ*^2^(*a*) the variance of *f*_*a*_, (2.4) generalizes to (see appendix A for the proof)

**Figure 19.**
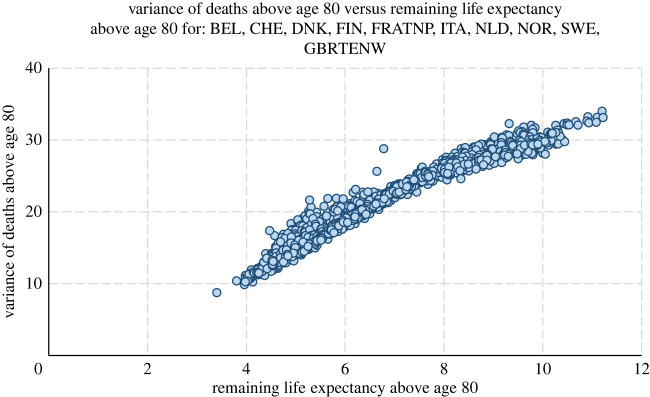
Plots of the variance of the ages at death of those surviving to at least 80 years of age (left axis, blue data) and the skewness of the age greater than or equal to 80 deaths distribution (right axis, red data) versus the remaining life expectancy for females and males in the 10 countries in the Human Mortality Database [23] with the oldest data (3343 yearly life tables for males and females spanning 1751–2016; see table 2 in appendix D for more information): Belgium (BEL), Switzerland (CHE), Denmark (DNK), Finland (FIN), France (total population; FRATNP), Italy (ITA), The Netherlands (NLD), Norway (NOR), Sweden (SWE) and the UK (England and Wales, total population; GBRTENW).

5.1 $$0\leq \sigma^{2}(a)\leq e(a)[(\omega -a)-e(a)],$$

where *e*(*a*) is the remaining life expectancy at age *a*. Thus, all of our arguments and results from §2 generalize, and the key takeaway is that, as before, the variance curves in these ‘*a*-truncated’ settings are again bounded by the right-hand side of (5.1), a quadratic function of *e*(*a*). (We note that the *a* = 0 case of (5.1) is (2.4).) Thus, (5.1) says that for those surviving to age *a*, the variance in the associated *a*-truncated deaths distribution again has a maximum value and again tends to zero as *e*(*a*) tends to 0 or *ω* − *a*. This implies, once more, the existence of non-longevous states (variance and life expectancy positively correlated) when the remaining life expectancy, *e*(*a*), is small; and longevous states (variance and life expectancy negatively correlated) when the remaining life expectancy, *e*(*a*), is large. The latter we have already illustrated in §4 for the *a* = 0 special case of (5.1); the former we illustrate in figure 19 in appendix D, which shows that, for those surviving to at least age 80 years in the same 10 countries studied in figure 3, the variance in the age of death and the remaining life expectancy are positively correlated. The implication of (5.1) for this age group is that, as *e*(80) continues to rise, said variance will eventually peak and begin to fall, culminating in a longevous state for those aged 80+. (One can already see the variance curve concavity developing by comparing the e(80)⪅8 and e(80)⪆8 regions of the data in figure 19.)

The second extension of our results herein follows from the generality of theorems 2.1–2.4. This generality, along with the empirical studies in §4, suggests to us that our three Variance Curve Hypotheses may hold for other species. (Indeed, for any species whose deaths distributions are modelled by distributions with finite support (e.g. a beta distribution) our results herein apply.) Support for this assertion in animal species, particularly mammals, comes from the fast–slow continuum, a well-known regularity in animals [24], as well as the well-known body size–longevity correlation—that short-lived animals tend to be small and die young while long-lived animals tend to be large and have long lifespans [34]. This body size–longevity correlation implies low uncertainty in the age at death at both extremes of longevity, and evolutionary senescence theory [35,36] implies that as short-lived species’ survivorship improves their rates of ageing slow [37], implying greater uncertainty in the age at death and hence an increasing variance function. These suggest variance curves for animal species that follow at least some aspects of our Variance Curve Correlation and Quantified Type Hypotheses. We therefore conjecture that our four hypotheses hold beyond just human populations. These are at present just conjectures; further empirical studies may uncover evidence for them, or they may identify taxa whose variance curves deviate from those predicted by our hypotheses. (Future work in this direction could probe theorems 2.4 and 2.5 for insights, much like we did when we discussed figure 2*e*,*f*.) Future work could also study the biodemographic drivers for either the robustness of our hypotheses, or the deviation from them for specific taxa. (We have suggested potential drivers of our hypotheses above, but further investigation is needed.) Finally, being sensitive to the fact *ω* is species-specific, future work could undertake a comparative study of inter-species variance curves. We conjecture there that at some taxonomic depth (e.g. genus) all encompassed species’ variance curves share similar features.

We end with a return to the discussion of the importance of our assumptions to our theoretical results. As we have reiterated throughout, what we have established here we have done so only for populations with finite maximum lifespans. Theorem 2.1 required this assumption, as did some of the deductions we made later regarding the existence of maximal values of variance curves. As we stressed at the start of §2, whether—and to what extent—our theoretical results hold when the finite *ω* assumption is replaced by another assumption on *ω* is an open question. We point out here that this other assumption need not be *ω* = ∞. For example, perhaps *ω* can be replaced with *ω*(*t*), a time-dependent yet still finite maximum lifespan. Or perhaps one can explore alternative ways of conceptualizing the notion of a finite maximum lifespan (see e.g. [38] and references therein, and also [19]). We suspect—but have no proof (yet)—that the theoretical results presented herein would carry over into these settings. (We note in passing that [19] shows that the (suitably) normalized Gini index converges to the classical Gini index as *ω* → ∞, meaning that perhaps for some lifespan inequality metrics the finite versus infinite *ω* question need not pose problems.) We are less optimistic—though not overtly pessimistic—about the *ω* = ∞ assumption. The reason is that, for example, when *ω* = ∞ and one considers the constant mortality case, *μ*(*x*) = *a* (where *a* > 0), then *f*(*x*) = *ae*^−*ax*^ and $\sigma^{2}(e_{0})=e_{0}^{2}$, a strictly increasing function with no maximum value. There are thus instances in the *ω* = ∞ setting where our theoretical results from the *ω* finite setting do not carry over. However, the constant mortality model is just one of many parametric mortality models studied in theoretical demography; whether or not our theoretical results hold in the *ω* = ∞ for other well-known parametric mortality models (e.g. Gompertz, Siler, etc.) remains to be seen. Such an investigation is beyond the scope of this article, however; this is yet another potential direction to explore in future work. Finally, let us remark on our four hypotheses. Unlike our theorems—which, as our proofs show, follow deductively from the assumptions we have made—our four Variance Curve Hypotheses are hypotheses. This means that even under the finite *ω* assumption there may exist populations which violate some (or all) those hypotheses. For example, there may exist finite *ω* mortality models—or empirical data on real-world populations—that generate variance curves which violate some or all of our hypotheses. The former type of challenge to our results would represent a theoretical challenge to our hypotheses—for example, a parametric mortality model satisfying theorem 2.1 and reasonably accurately modelling real-world populations yet predicting variance curves contradicting our hypotheses. The latter type of challenge would arise from identifying a real-world population whose variance curve likewise contradicts our hypotheses (e.g. a variance curve with two or more local maxima). While we have provided empirical evidence for our hypotheses in human populations and discussed above why we suspect they hold for non-human populations as well, we cannot rule out these possible challenges to our findings. Whether our hypotheses hold more generally—and why—remains to be determined. We invite researchers to probe these questions and investigate the universality of our hypotheses and their potential biological, ecological and demographic drivers.

## Data Availability

The data that support the findings of this study are openly available as part of the Human Mortality Database: https://www.mortality.org/ [23].

## Appendix A. Proofs

Proof of theorem 2.1. That *σ*^2^ ≥ 0 follows from (2.3) and the fact that (*x* − *e*_0_)^2^*f*(*x*) ≥ 0 for all *x* ∈ [0, *ω*]. To establish the upper bound in (2.4), we note first that since *x* ≤ *ω*,

$$\int_{0}^{\omega }x^{2}f\;(x)\;dx=\int_{0}^{\omega }x[xf\;(x)]\;dx\leq \int_{0}^{\omega }\omega [xf\;(x)]\;dx=\omega \int_{0}^{\omega }xf\;(x)\;dx=\omega e_{0},$$

using (2.2). Finally, using (2.3) yields

$$\sigma^{2}=\int_{0}^{\omega }x^{2}f\;(x)\;dx-e_{0}^{2}\leq \omega e_{0}-e_{0}^{2}=\frac{\omega^{2}}{4}-{\left(e_{0}-\frac{\omega }{2}\right)}^{2},$$

the latter following by completing the square. We note in passing that, devoid of the demographic context used herein, the inequality above is known to statisticians as the Bhatia–Davis inequality [39]. And while there are numerous refinements of this inequality (see [40] for a detailed summary), the formulation in (2.4) will suffice for our purposes. ▪

Proof of theorem 2.4. Using (2.1) in (2.3) and then integrating by parts yields

A 1 $$\begin{array}{rl} \sigma^{2} & =\int_{0}^{\omega }x^{2}f\;(x)\;dx-e_{0}^{2}\\ & =-\int_{0}^{\omega }x^{2}s^{\prime }(x)\;dx-e_{0}^{2}\\ & =2\int_{0}^{\omega }xs(x)\;dx-e_{0}^{2}\\ & =2e_{0}\bar{x}-e_{0}^{2}, \end{array}$$

where $\bar{x}=(1/e_{0})\int_{0}^{\omega }xs(x)\;dx$ is the mean age in the stationary population. (Above and hereafter, parameters without letter subscripts (e.g. *e*_0_ versus *e*_0,*g*_) are calculated relative to the deaths distribution *f*.) Similarly,

$$\sigma_{g}^{2}=2e_{0,g}{\bar{x}}_{g}-e_{0,g}^{2}.$$

Noting that Δ*e*_0_ = *e*_0,*g*_ − *e*_0_ and $\Delta \bar{x}={\bar{x}}_{g}-\bar{x}$, we have

A 2 $$\begin{array}{rl} \Delta (\sigma^{2})=\sigma_{g}^{2}-\sigma^{2} & =2e_{0,g}{\bar{x}}_{g}-e_{0,g}^{2}-(2e_{0}\bar{x}-e_{0}^{2})\\ & =2(e_{0}+\Delta e_{0})(\bar{x}+\Delta \bar{x})-{\left(e_{0}+\Delta e_{0}\right)}^{2}-2e_{0}\bar{x}+e_{0}^{2}\\ & =2e_{0}\Delta \bar{x}+2\bar{x}\Delta e_{0}+2\Delta e_{0}\Delta \bar{x}-2e_{0}\Delta e_{0}-{\left(\Delta e_{0}\right)}^{2}\\ & =2(\bar{x}-e_{0})\Delta e_{0}+2e_{0}\Delta \bar{x}+2\Delta e_{0}\Delta \bar{x}-{\left(\Delta e_{0}\right)}^{2}. \end{array}$$

Assuming now that Δ*e*_0_ ≠ 0,

A 3 $$\begin{array}{rl} \frac{\Delta (\sigma^{2})}{\Delta e_{0}} & =2(\bar{x}-e_{0})+2e_{0}\frac{\Delta \bar{x}}{\Delta e_{0}}+2\Delta \bar{x}-\Delta e_{0}\\ & =2e_{0}\left\{\frac{\Delta \bar{x}}{\Delta e_{0}}+\left(\frac{\bar{x}}{e_{0}}-1\right)+\frac{1}{2e_{0}}[2\Delta \bar{x}-\Delta e_{0}]\right\}. \end{array}$$

Now, we note that from (A 1),

$$\frac{\bar{x}}{e_{0}}=\frac{1}{2}\left(\frac{\sigma^{2}}{e_{0}^{2}}+1\right)=\frac{1}{2}(v^{2}+1),$$

where *v* = *σ*/*e*_0_ is the coefficient of variation. Using this in (A 3) yields

A 4 $$\frac{\Delta (\sigma^{2})}{\Delta e_{0}}=2e_{0}\left\{\frac{\Delta \bar{x}}{\Delta e_{0}}-\frac{1}{2}(1-v^{2})+\frac{1}{2e_{0}}[2\Delta \bar{x}-\Delta e_{0}]\right\},$$

which is (2.8). And since *e*_0_ ≥ 0, the left- and right-hand sides of this equation have the same signs, from which the rest of the first part of the theorem follows. To prove the second part we simply take the limit as Δ*e*_0_ → 0 of (A 4) and use the fact that Δ*e*_0_ → 0 implies $\Delta \bar{x}\to 0$. In this limit, the bracketed term in (A 4) tends to zero and what remains is precisely (2.9). The rest of the theorem then follows again from noting that both the left-hand side of (2.9) and *J* have the same sign, since *e*_0_ ≥ 0. For completeness, we make two additional remarks on this last portion of the proof. First, we note that an alternative proof of (2.9) follows from differentiating (A 1) with respect to *e*_0_ and solving the result for $d\bar{x}/de_{0}$. Lastly, we note that since $d\sigma^{2}/de_{0}={\dot{\sigma }}^{2}(t)/{\dot{e}}_{0}(t)$, where dots denote differentiation with respect to *t*, our proof does not assume that *σ*^2^ is a function of *e*_0_. ▪

Proof of theorem 2.5. From (2.3) we have:

$$\sigma^{2}[f+\varepsilon \nu ]=\int_{0}^{\omega }x^{2}[f\;(x)+\varepsilon \nu (x)]\;dx-{\left(\int_{0}^{\omega }x[f\;(x)+\varepsilon \nu (x)]\;dx\right)}^{2}.$$

We then obtain

$$\frac{d}{d\varepsilon }\sigma^{2}[f+\varepsilon \nu ]=\int_{0}^{\omega }x^{2}\nu (x)\;dx-2\left(\int_{0}^{\omega }x[f\;(x)+\varepsilon \nu (x)]\;dx\right)\left(\int_{0}^{\omega }x\nu (x)\;dx\right).$$

Setting *ɛ* = 0 and using (2.2) then yields (2.11). ▪

Proof of (5.1). We start from the definitions

$$\sigma^{2}(a)=\int_{a}^{\omega }{\left(x-a\right)}^{2}f_{a}(x)\;dx-{\left[e(a)\right]}^{2},\;\text{where}\;f_{a}(x)=\frac{\;f\;(x)}{\int_{a}^{\omega }f\;(x)\;dx}\;$$

and

$$e(a)=\int_{a}^{\omega }(x-a)f_{a}(x)\;dx,$$

see [17]. Then, following the proof of theorem 2.1,

$$\begin{array}{rl} \sigma^{2}(a) & =\int_{a}^{\omega }(x-a)[(x-a)f_{a}(x)]\;dx-{\left[e(a)\right]}^{2}\\ & \leq (\omega -a)\int_{a}^{\omega }(x-a)f_{a}(x)\;dx-{\left[e(a)\right]}^{2}\\ & =(\omega -a)e(a)-{\left[e(a)\right]}^{2}, \end{array}$$

which simplifies to (5.1). ▪

## Appendix B. Theoretical example: the hyperbolic mortality case

Consider the hyperbolic mortality model,

B 1 $$\mu (x)=\frac{a}{\omega -x},\;f\;(x)=\frac{a}{\omega }{\left(1-\frac{x}{\omega }\right)}^{a-1},\;a>0,\;0\leq x<\omega ,$$

whose associated survival function is

B 2 $$s(x)={\left(1-\frac{x}{\omega }\right)}^{a},\;a>0,\;0\leq x\leq \omega .$$

We then have the following result.

Proposition B.1. *For the hyperbolic mortality model* (*B* 1),

B 3 $$e_{0}=\frac{\omega }{a+1},\;\sigma^{2}=e_{0}^{2}\left(\frac{\omega -e_{0}}{\omega +e_{0}}\right).$$

Proof. We first calculate *e*_0_ (from (2.2)):

B 4 $$e_{0}=\int_{0}^{\omega }{\left(1-\frac{x}{\omega }\right)}^{a}\;dx=-\frac{\omega }{a+1}{\left[{\left(1-\frac{x}{\omega }\right)}^{a+1}\right]}_{0}^{\omega }=\frac{\omega }{a+1}.$$

For the second part, we note that

B 5 $$f\;(x)=-s^{\prime }(x)=\frac{a}{\omega }{\left(1-\frac{x}{\omega }\right)}^{a-1}.$$

Thus,

B 6 $$\begin{array}{rl} \int_{0}^{\omega }x^{2}f\;(x)\;dx & =\frac{a}{\omega }\int_{0}^{\omega }x^{2}{\left(1-\frac{x}{\omega }\right)}^{a-1}\;dx\\ & =-\frac{1}{\left(a+1)(a+2\right)}{\left[{\left(1-\frac{x}{\omega }\right)}^{a}(2a\omega x+a(a+1)x^{2}+2\omega^{2})\right]}_{0}^{\omega }\\ & =\frac{2\omega^{2}}{\left(a+1)(a+2\right)}. \end{array}$$

It follows, using (B 4), that

B 7 $$\sigma^{2}=\int_{0}^{\omega }x^{2}f\;(x)\;dx-e_{0}^{2}=\frac{2\omega^{2}}{\left(a+1)(a+2\right)}-\frac{\omega^{2}}{{\left(a+1\right)}^{2}}=e_{0}^{2}\left(\frac{\omega -e_{0}}{\omega +e_{0}}\right),$$

which yields the second equation in (B 3). ▪

The survival curves (B 2) associated with the hyperbolic mortality case capture the basic features one sees in real-world populations where *ω* has empirically been found to be finite. Figure 5*a* shows the survival function associated with five different hyperbolic mortality functions; for small *e*_0_-values (which correspond to large *a*-values because of (B 3)) the curves are textbook Type III curves (the red curves in the figure) characteristic of populations with high early-age mortality. As life expectancy increases, survival resembles the more characteristic Type II curves (the grey curve in the figure), and eventually textbook Type I curves (the solid blue curves in the figure). The associated variance curve documenting the relation between *σ*^2^ and *e*_0_, the function of *e*_0_ given in (B 3), is graphed in figure 5*b* (in red). We note that it adheres to the upper bound established in theorem 2.1 (graphed in black in the figure).

Next, let us illustrate theorem 2.4, specifically (2.9). Differentiating the *σ*^2^(*e*_0_) function from (B 3) yields

B 8 $$\frac{d\sigma^{2}}{de_{0}}=-\frac{2e_{0}}{{\left(\omega +e_{0}\right)}^{2}}[e_{0}^{2}+e_{0}\omega -\omega^{2}]=2e_{0}\left\{-\frac{e_{0}^{2}+e_{0}\omega -\omega^{2}}{{\left(\omega +e_{0}\right)}^{2}}\right\}.$$

We now show that the term in braces is precisely *J* from (2.7). To do so we first express $\bar{x}$ as a function of *e*_0_ and then differentiate that expression with respect to *e*_0_. Starting with $\bar{x}=(\sigma^{2}+e_{0}^{2})/2e_{0}$ (cf., (A 1)) and substituting in *σ*^2^(*e*_0_) from (B 3) yields

$$\bar{x}(e_{0})=\frac{e_{0}}{2}+\frac{1}{2e_{0}}\left[e_{0}^{2}\left(\frac{\omega -e_{0}}{\omega +e_{0}}\right)\right]=\frac{e_{0}}{2}\left[1+\frac{\omega -e_{0}}{\omega +e_{0}}\right].$$

Differentiating this with respect to *e*_0_ yields

$$\frac{d\bar{x}}{de_{0}}=\frac{\omega^{2}}{{\left(\omega +e_{0}\right)}^{2}}\;⟹\;\frac{d\bar{x}}{de_{0}}-\frac{1}{2}(1-v^{2})=\frac{\omega^{2}}{{\left(\omega +e_{0}\right)}^{2}}-\frac{1}{2}\left[\left(1-\frac{\omega -e_{0}}{\omega +e_{0}}\right)\right]=-\frac{e_{0}^{2}+e_{0}\omega -\omega^{2}}{{\left(\omega +e_{0}\right)}^{2}}.$$

Thus, (B 8) can be written as

$$\frac{d\sigma^{2}}{de_{0}}=2e_{0}\left[\frac{d\bar{x}}{de_{0}}-\frac{1}{2}(1-v^{2})\right],$$

which reproduces (2.9).

Let us next illustrate how theorem 2.4 can be leveraged to obtain information about the concavity of variance curves. To do so, we note that from (2.9),

$$\frac{d^{2}\sigma^{2}}{de_{0}^{2}}=2\left[J+e_{0}\frac{dJ}{de_{0}}\right].$$

The concavity of the variance curve, therefore, depends on the sign of the bracketed term. Furthermore, when that term vanishes we obtain candidate inflection points. In the context of the hyperbolic mortality case, from the above computations,

$$J=\frac{d\bar{x}}{de_{0}}-\frac{1}{2}(1-v^{2})=-\frac{e_{0}^{2}+e_{0}\omega -\omega^{2}}{{\left(\omega +e_{0}\right)}^{2}}.$$

Setting *J* + *e*_0_(d*J*/d*e*_0_) = 0 then yields

$$e_{0}^{2}+e_{0}\omega -\omega^{2}+e_{0}\omega \frac{3\omega +e_{0}}{\omega +e_{0}}=0.$$

Solving this for *e*_0_ yields $e_{0}=(3\sqrt{2}-1)\omega$ as the only real solution. This is precisely the location of the (only) inflection point of the variance curve associated with the hyperbolic mortality case (the red curve in figure 5*b*).

Finally, let us now illustrate theorem 2.5. To do so we will return to the associated deaths distribution *f* from (B 1) and consider perturbations of that distribution by replacing *a* with *a* + *ɛ*. This yields the following family of perturbed deaths distributions:

B 9 $$f_{\varepsilon }(x)=\frac{a+\varepsilon }{\omega }{\left(1-\frac{x}{\omega }\right)}^{a+\varepsilon -1}=f\;(x)+\varepsilon \left[{\left(1-\frac{x}{\omega }\right)}^{a-1}\frac{1+a\ln(1-(x/\omega ))}{w}\right]+O(\varepsilon^{2}),$$

where *O*(*ɛ*^2^) is Landau notation indicating that the remaining terms in this expansion are of order at least *ɛ*^2^. The term in brackets is *ν*(*x*), in the notation of theorem 2.5. (In figure 1*c*, the black and red curves are the deaths distributions *f* (from (B 1)) for *a* = 1.5 and *a* = 1.6, respectively, and the blue curve is the difference of those two distributions, which approximates *v*(*x*).) Straightforward calculations then yield

$$-2e_{0}\delta e_{0}[f;\nu ]=\frac{2\omega^{2}}{{\left(a+1\right)}^{3}}\;\mathrm{and}\;\int_{0}^{\omega }x^{2}\nu (x)\;dx=-\frac{2\omega^{2}(2a+3)}{{\left(a+1\right)}^{2}{\left(a+2\right)}^{2}}.$$

Thus,

B 10 $$\delta (\sigma^{2})[f;\nu ]=\frac{2\omega^{2}}{{\left(a+1\right)}^{3}}-\frac{2\omega^{2}(2a+3)}{{\left(a+1\right)}^{2}{\left(a+2\right)}^{2}}=-\frac{2(a^{2}+a-1)\omega^{2}}{{\left(a+1\right)}^{3}{\left(a+2\right)}^{2}}.$$

If we now return to (B 3) and express *σ*^2^ in terms of *ɛ* we obtain

$$\sigma^{2}(\varepsilon )={\left(\frac{\omega }{a+\varepsilon +1}\right)}^{2}\left(\frac{\omega -(\omega /(a+\varepsilon +1))}{\omega +(\omega /(a+\varepsilon +1))}\right).$$

Expanding this in terms of *ɛ* yields

B 11 $$\begin{array}{rl} \sigma^{2}(\varepsilon ) & =\sigma^{2}(0)+\varepsilon \left[-\frac{2(a^{2}+a-1)\omega^{2}}{{\left(a+1\right)}^{3}{\left(a+2\right)}^{2}}\right]+O(\varepsilon^{2})\\ & =\sigma^{2}(0)+\varepsilon \delta (\sigma^{2})[f;\nu ]+O(\varepsilon^{2}), \end{array}$$

after recalling (B 10). Thus, *δ*(*σ*^2^)[*f*; *ν*] indeed measures the change in *σ*^2^ to first order in *ɛ*.

**B.1. Illustrating the hypotheses from §3**

The two most straightforward verifications are of the Correlation and Pattern Hypotheses. Indeed, as figure 5*b* shows, the variance curve in the hyperbolic mortality case is a fundamental variance curve, and thus automatically satisfies the Correlation Hypothesis.

To discuss the Type Hypothesis we return to figure 5. As figure 5*a* shows—and (B 2) confirms—the survival curves for *a* > 1 are the convex curves below the grey curve in the figure (the two red curves shown are examples), the grey line is the *a* = 1 survival curve, and for *a* < 1 the survival curves are the concave curves above the grey curve in the figure (the two blue curves shown are examples). We can thus safely classify *a* ≫ 1 as Type III curves, *a* = 1 as a Type II curve, and *a* ≪ 1 as Type I curves. For a⪆1 and a⪅1, however, we run into the ambiguity mentioned in §3. Are the survival curves corresponding to *a* = 1 ± *δ* Type II, III or I? Regardless of the answer, if we consider a hypothetical population in an initially Type III state that evolves over time into a Type II and then Type I state, the corresponding progression of *a*-values is a decrease from *a* ≫ 1 to *a* ≈ 1 to eventually *a* ≪ 1, respectively. In terms of the variance curve, as figure 6 shows—which overlays the *a*-values corresponding to *e*_0_ (from (B 3)) onto the variance curve in figure 5*b*—this hypothetical type evolution would trace out the variance curve from left to right, so that the correlation between the variance in age at death and life expectancy would change sequentially from positive to zero to negative. For the red-coloured portion of the curve we have *a* > 1, and thus those *a* > 1 Type III survival curve states are associated with a positive variance–life expectancy correlation. For the blue-coloured portion of the curve we have *a* < 0.37, and thus those *a* < 1 Type I survival curve states are associated with a negative variance–life expectancy correlation. This verifies all but the Type II portion of the Variance Curve Type Hypothesis. Whether the hypothesis holds for the Type II states depends on the aforementioned ambiguity in classifying the Type II states. If one considers survival curves with 0.37 ≤ *a* ≤ 1 as Type II then the corresponding portion of the variance curve—the grey portion of the curve in figure 6—indicates approximately no correlation between the variance of ages at death and life expectancy, verifying the final chunk of the Type Hypothesis. Other definitions of Type II, however, will change this conclusion.

To illustrate the Quantified Type Hypothesis we first calculate the skewness ${\tilde{\mu }}_{3}$ of the deaths distribution *f* in (B 1) as a function of *a*,

B 12 $${\tilde{\mu }}_{3}=\frac{1}{\sigma^{3}}\int_{0}^{\omega }{\left(x-e_{0}\right)}^{3}f\;(x)\;dx=\frac{1}{\sigma^{3}}\left(\frac{2a(a-1)\omega^{3}}{{\left(a+1\right)}^{3}(a+2)(a+3)}\right)=\frac{2(a-1)}{a+3}\sqrt{\frac{a+2}{a}},$$

where we made use of (B 3) to express *e*_0_ and *σ*^2^ in terms of *a*. This result tells us that the hyperbolic mortality deaths distribution *f* (from (B 1)) is positively skewed for *a* > 1, is skew zero for *a* = 1, and is negatively skewed for *a* < 1. These are the same *a*-value ranges that delineated the survival curve types earlier. Thus we reach the same conclusions as before, confirming the Quantified Type Hypothesis, if Type II survival curves are again defined as survival curves corresponding to 0.37 ≤ *a* ≤ 1.

## Appendix C. Data analysis

We conducted all analyses in the statistical software R [41]. We estimated all quantities involving definite integrals via the trapezoidal rule. For example, to estimate *σ*^2^ from (2.3) we applied the trapezoidal rule to $\int_{0}^{\omega }x^{2}\;f\;(x)\;dx$, where *x* corresponds to age and *f*(*x*) is the life-table distribution of ages at death in single years of age, and to *e*_0_, as defined by (2.2).

## Appendix D

**Table 2.** Table of countries in the HMD [23] dataset we used in the empirical analysis in §4. *N* corresponds to the number of period life tables used (males and females combined).

HMD abbreviation	country	start year	end year	*N*
BEL	Belgium	1841	2016	169
CHE	Switzerland	1876	2016	280
DNK	Denmark	1835	2016	364
FIN	Finland	1878	2016	278
FRATNP	France	1816	2016	402
GBRTENW	England and Wales	1841	2016	352
ITA	Italy	1872	2016	290
NLD	The Netherlands	1850	2016	334
NOR	Norway	1846	2016	342
SWE	Sweden	1751	2016	532
